# NMR solution and X-ray crystal structures of a DNA molecule containing both right- and left-handed parallel-stranded G-quadruplexes

**DOI:** 10.1093/nar/gkz349

**Published:** 2019-06-19

**Authors:** Fernaldo Richtia Winnerdy, Blaž Bakalar, Arijit Maity, J Jeya Vandana, Yves Mechulam, Emmanuelle Schmitt, Anh Tuân Phan

**Affiliations:** 1 School of Physical and Mathematical Sciences, Nanyang Technological University, Singapore 637371, Singapore; 2 Laboratoire de Biochimie, UMR 7654, CNRS, Ecole Polytechnique, Palaiseau 91128, France

## Abstract

Analogous to the B- and Z-DNA structures in double-helix DNA, there exist both right- and left-handed quadruple-helix (G-quadruplex) DNA. Numerous conformations of right-handed and a few left-handed G-quadruplexes were previously observed, yet they were always identified separately. Here, we present the NMR solution and X-ray crystal structures of a right- and left-handed hybrid G-quadruplex. The structure reveals a stacking interaction between two G-quadruplex blocks with different helical orientations and displays features of both right- and left-handed G-quadruplexes. An analysis of loop mutations suggests that single-nucleotide loops are preferred or even required for the left-handed G-quadruplex formation. The discovery of a right- and left-handed hybrid G-quadruplex further expands the polymorphism of G-quadruplexes and is potentially useful in designing a left-to-right junction in G-quadruplex engineering.

## INTRODUCTION

The right- and left-handed helical progressions in double helix DNA were discovered in the structure of B-DNA and Z-DNA respectively (1,2). The temporary formation of Z-DNA near promoter regions was shown to affect transcription processes (3,4), in which state the double helix-DNA would contain two B- to Z-DNA junctions. The crystal structure of a B to Z-DNA junction was previously examined, proving the coexistence of the two helical states in one double-helix chain (5). The interconversion between right- and left-handed helical structures in double-helix DNA is believed to hold a vital role in regulating many biological processes (6) and proteins have been shown to have high affinity specific binding toward Z-DNA (7,8). In this study, the concept of DNA handedness compatibility is extended to G-quadruplexes (G4)—four-stranded non-canonical secondary structures of DNA, which can also be specifically recognized by a range of proteins and/or ligands (9–12).

The core of a G4 structure normally consists of the stacking of two or more G-tetrads—a square planar arrangement of four guanine bases—stabilized by cations (13,14). G4 structures have been studied for decades resulting in the discovery of various G-quadruplex topologies, differing in terms of backbone orientations, number of G-tetrads, molecularity, types of connecting loops, types of ions, among others (15–35). However, until recently, all discovered G4 structures were based on a right-handed helical progression. The first left-handed G4 structure of natural DNA was discovered in the form of *Z-G4*, a four-layered structure comprising of two stacking left-handed parallel G4 blocks (36). Several unique structural characteristics of left-handed G4 beyond its tetrad progression include the capping property of single-residue thymine loops and different sugar-phosphate backbone dihedral angles between the core guanine residues (36).

A 12-nt minimal motif for left-handed G4 formation was recently identified (37), from which a dimeric left-handed G4 structure was resolved. The original *Z-G4* sequence can be separated into two halves: the first half is a G-rich sequence which on its own would fold into a right-handed parallel G4 (designated as *Block1*, 5′-TGGTGGTGGTGGTT-3′), and the second half is the aforementioned minimal motif (designated as *Block2*, 5′-GTGGTGGTGGTG-3′) (Supplementary Figure S1). Hence, the *Z-G4* structure effectively demonstrated the ability of the *Block2* sequence to alter the structure of *Block1* into a left-handed structure, notwithstanding its original conformation. Substitutions of some T nucleotide loops of *Block1* by A or C did not abolish the left-handedness of the sequence (37), showing that *Block2* could drive various adjacent sequences into a left-handed G4 conformation. We attempted to study the conformational switching ability of *Block2* for other G4 forms by attaching it to the thrombin-binding aptamer (*TBA*) sequence—a 15-mer G-rich sequence, which forms a right-handed anti-parallel G4 structure (38) and can bind specifically to exosite I of human alpha-thrombin (39). Surprisingly, we found that connecting *TBA* and *Block2* sequences resulted in a structure containing both right- and left-handed parallel G4 on top of each other. In both NMR solution and X-ray crystal structures, the *TBA* sequence is found to adopt a right-handed parallel conformation, while the *Block2* sequence remains in its left-handed parallel conformation. The conformational altering property of *Block2* is found to be more delicate and sequence-specific as it converts the *TBA* sequence from an anti-parallel to parallel right-handed conformation. We utilize the crystal structure of the combined sequence to examine the structural characteristics of both the individual right- and left-handed blocks, as well as the junction interface. The coexistence of both types of helical progression in a single G4 structure not only confirms the occurrence of right- to left-handed helical junctions in G4, but also further adds on to the repertoire of widely diverse G4s.

## MATERIALS AND METHODS

### Sample preparation

Unlabeled DNA oligonucleotides were purchased from IDT with a standard desalting purification in the scale of 100 nmol to 1 μmol. Sample purity measured with ESI-MS was >99%. Site-specific labeled DNA oligonucleotides were chemically synthesized on an ABI 394 DNA synthesizer using products from Glen Research and Cambridge Isotope Laboratories, then purified following the protocol from Glen Research. The samples (concentration, 0.2−2 mM) were dialyzed successively against water, 25 mM KCl, and water again. DNA oligonucleotides were frozen, lyophilized, and dissolved in buffer containing 70 mM KCl and 20 mM KPi (pH 7) buffer. The DNA concentration was expressed in strand molarity using the nearest neighbor approximation for the 260 nm molar extinction coefficient of the unfolded species.

### Circular dichroism

Circular dichroism (CD) spectra were recorded on a JASCO-815 spectropolarimeter using 1-cm path length quartz cuvettes at 20°C. Scans from 220 to 320 nm were performed with a scanning speed of 100 nm/min, 1-nm data pitch, 1-nm bandwidth and 1 s digital integration time (DIT). For each measurement, an average of three scans was taken, the spectral contribution of the buffer was subtracted and data were zero-corrected at 320 nm. DNA samples with concentrations of 3–8 μM were prepared in a buffer containing 70 mM KCl, 20 mM KPi (pH 7). Molar ellipticity of the CD spectra was calculated using the DNA concentration derived from the sample absorbance at 260 nm.

### NMR spectroscopy

NMR experiments were performed at 25°C on Bruker Avance II and III spectrometers operating at 600 and 800 MHz respectively. The DNA concentration for NMR experiments was typically 0.1−1.5 mM in 70 mM KCl, 20 mM KPi (pH 7) at 25°C, unless otherwise specified. Assignment of the imino protons of guanine residues was obtained by ^15^N-filtered experiments using 2% site-specific labelled samples. Assignments of guanine aromatic protons were obtained via long-range through-bond correlation between imino and aromatic protons. Spectra analyses were performed using the Topspin 3.5 (Bruker) and SPARKY 3.1 (40) softwares.

### NMR structure calculations

#### NOE distance restraints

Inter-proton distances for *TBA-TT-Block2* were obtained from NOESY experiments performed in H_2_O and D_2_O at various mixing times (100, 200 and 300 ms). For non-exchangeable protons, the peaks were classified as strong, medium, medium-weak and weak corresponding to the distance restraints of 2.7 ± 0.8, 3.8 ± 0.9, 4.6 ± 1.2 and 5.5 ± 1.7 Å respectively. Distances from exchangeable protons were classified as strong, medium and weak corresponding to the distance restraints of 4.0 ± 1.2, 4.8 ± 1.4 and 5.5 ± 1.7 Å respectively. Distance restraints involving thymine methyl groups were directed towards the methyl carbon with strong, medium and weak NOEs being restrained to 4.0 ± 1.2, 4.8 ± 1.4 and 5.5 ± 2.2 Å respectively.

#### Dihedral restraints

Dihedral angle restraints were imposed to the dihedral angle formed by O4′–C1′–N9–C4 of guanine residues. *Anti*-guanine residues were restricted to an angle of (240 ± 70)° or (240 ± 40)° for the outer tetrad and inner tetrad guanines respectively.

#### Hydrogen-bond restraints

Hoogsteen hydrogen bonds between guanines were restrained using H21–N7, N2–N7, H1–O6 and N1–O6 distances, which were set to 2.0 ± 0.2 Å, 2.9 ± 0.3, 2.0 ± 0.2 and 2.9 ± 0.3 Å respectively.

#### Planarity restraints

Planarity restraints were used for the G2•G6•G11•G15, G1•G5•G10•G14, G18•G21•G24•G27 and G20•G23•G26•G29 tetrads.

#### Distance-geometry simulated annealing

Initial extended conformation of *TBA-TT-Block2* sequence was generated using the XPLOR-NIH (41) program by supplying the available standard nucleic acid topology and parameter tables. Each system was then subjected to distance-geometry simulated annealing by incorporating distance, dihedral, hydrogen-bond and planarity restraints. One hundred structures were generated and subjected to further refinement.

#### Distance-restrained molecular dynamics refinement

The 100 structures obtained from each simulated annealing step were refined with a distance-restrained molecular dynamics protocol incorporating all distance restraints. The system was heated from 300 to 1000 K in 14 ps and allowed to equilibrate for 6 ps, during which force constants for the distance restraints were kept at 2 kcal mol^−1^.Å^−2^. The force constants for non-exchangeable proton and exchangeable proton restraints were then increased to 16 kcal mol^−1^.Å^−2^ and 8 kcal mol^−1^.Å^−2^ respectively in 20 ps before another equilibration at 1000 K for 50 ps. Next, the system was cooled down to 300 K in 42 ps, after which an equilibration was performed for 18 ps. Coordinates of the molecule were saved every 0.5 ps during the last 10.0 ps and averaged. The average structure obtained was then subjected to minimization until the gradient of energy was <0.1 kcal mol^−1^. Dihedral (50 kcal mol^−1^ rad^−2^) and planarity (1 kcal mol^−1^ Å^−2^ for tetrads) restraints were maintained throughout the course of refinement. Ten-lowest energy structures were generated.

### Crystallization

*TBA-T-Block2* solution at a concentration of 1 mM was prepared in 100 mM potassium cacodylate buffer (pH 7). Prior to using for crystallization, the sample was annealed by heating at 95°C for 5 min followed by slowly cooling to room temperature. Initial screening for crystallization conditions was done at 24°C using Natrix 1 & 2 reagents set (Hampton Research) in a 96-well sitting drop vapor diffusion setup at both 2:1 and 1:1 sample-to-reagent proportions with the help of mosquito^®^ LCP (ttplabtech). Rod shaped crystals were found to form within 7–10 days under the following condition: 0.08 M potassium chloride, 0.04 M sodium cacodylate trihydrate pH 7.0, 60% (v/v) -2-methyl-2,4-pentanediol and 0.012 M spermine tetrahydrochloride. Crystals were flash frozen in liquid nitrogen before data collection.

### X-ray diffraction data collection and refinement

Crystal diffraction data were collected at the PROXIMA 1 beamline of the SOLEIL synchrotron, France. Native datasets were collected over 360° rotation ranges at 0.1° oscillation range (Table 2). Data was processed using the XDS software package (42). Initially, the data were processed in *P*2_1_ space group. Molecular replacement was done using the *Z-G4* crystal structure (PDB ID: 4U5M) as a search model to obtain initial phases. Two copies per asymmetric unit were found. Reconstruction in the molecular replacement density map clearly indicated the presence of one right-handed and one left-handed block. The model was iteratively built through cycles of refinement using Phenix (43,44) and manual rebuilding in Coot (45). However, the refinement could not be improved beyond *R*_work_ and *R*_free_ values of 23 and 27%, respectively. Moreover, strong signs of twinning were observed in the electron density of T residues from the right-handed block. The data were therefore further processed in the *P*1 space group. In order to increase completeness, data from two isomorphic crystals fished in the same crystallization drop were merged. The twin law -*h, k*, -*l* was used during the refinement procedure in Phenix. The structure was refined to final *R*_work_, *R*_free_ values of 16.42% and 20.19% respectively (Table 2) which contained four molecules in the asymmetric unit.

## RESULTS AND DISCUSSION

### The left-handed motif (*Block2*) converts the anti-parallel thrombin-binding aptamer (*TBA*) G-quadruplex to a new conformation

**Figure 1.**
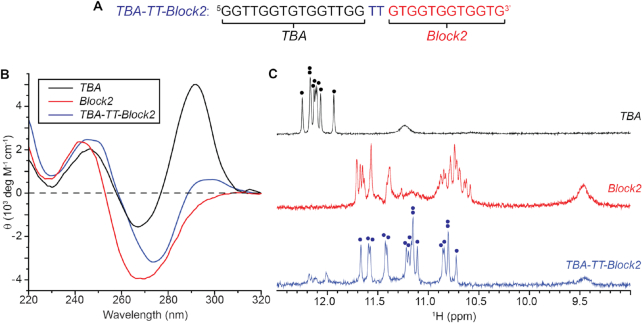
(**A**) Sequence, (**B**) CD and (**C**) NMR spectra of *TBA* (black), *Block2* (red) and *TBA-TT-Block2* (blue). The CD spectra of *TBA* and *Block2* resemble that of right-handed anti-parallel G4 and left-handed parallel G4 respectively, while a unique spectrum is observed for *TBA-TT-Block2*.

Circular dichroism and one-dimensional (1D) ^1^H NMR experiments of both the *TBA* and *Block2* sequences (Figure 1A) were performed in potassium buffer (70 mM KCl, 20 mM KPi, pH 7.0). The CD spectrum of *TBA* showed peaks at 245 and 295 nm, as well as a trough at 265 nm, typical of right-handed anti-parallel G4s. On the other hand, CD spectrum of *Block2* revealed a peak at 245 nm and a relatively larger trough at 270 nm, characteristic of left-handed parallel G4s (Figure 1B) (46–48). The 1D NMR spectrum of *TBA* displayed eight imino proton peaks at around 12 ppm, while the spectrum of *Block2* exhibit two groups of imino proton peaks centered at ∼11.5 and ∼10.5 ppm (Figure 1C). The clear distinction between two imino proton groups and the observation of amino proton peaks at ∼9.5 ppm in the NMR spectrum of *Block2* are consistent with characteristic NMR spectra of left-handed G4s found to date (36,37).

The merging of the *TBA* and *Block2* oligonucleotide sequences with a single or double thymine linkers (designated as *TBA-T-Block2* or *TBA-TT-Block2*, respectively) resulted in highly similar CD and NMR spectra (Supplementary Figure S2). Time-series measurements of *TBA-T-Block2* (Supplementary Figure S3) demonstrated slow evolution of its imino proton spectra. A sample of the *TBA-T-Block2* sequence was quenched (heated and quickly cooled) and immediately measured by NMR and CD spectroscopy. Initially, a *TBA*-like NMR spectrum, with eight peaks around 12 ppm, equivalent to that of *TBA* alone (38) was observed, which suggest that the anti-parallel structure is kinetically favorable. The initial CD spectrum also indicated an anti-parallel structure with two peaks at ∼245 and ∼295 nm as well as a trough at ∼265 nm. The sample was then observed for over 8 days with both techniques. NMR experiments revealed the disappearance of the initial set of peaks and appearance of a new set of 16 peaks (Supplementary Figure S3A), while CD experiments showed the evolution of the spectrum away from the *TBA* anti-parallel signature (Supplementary Figure S3B). These results clearly demonstrated the conversion of the initial anti-parallel G4 form of *TBA* towards a more suitable form that accommodated the rest of the sequence (from *Block2*) in the final structure.

The CD spectrum of *TBA-TT-Block2* after >8 days incubation showed a slight deviation from the typical characteristics of a parallel left-handed G4 fold, with an additional low-amplitude peak at ∼295 nm and shift of the negative peak from 270 to 275 nm. Its NMR spectrum showed 16 major well-resolved imino proton peaks at ∼10.5 to 12 ppm, along with amino proton peaks at 9.5 ppm (Figure 1). Both the spectroscopic techniques implied a certain similarity between the structure of *TBA-TT-Block2* to those of parallel left-handed G4s. However, deviation of NMR and CD characteristics, such as the absence of two distinct imino proton groups and emergence of a CD peak at ∼295 nm potentially suggested a different folding topology.

### NMR solution structure of *TBA-TT-Block2* reveals the formation of adjacent right- and left-handed G-quadruplex blocks

Guanine imino proton resonance assignment of *TBA-TT-Block2* was obtained partly by the site-specific 2% ^15^N enrichment method (G1–G15, Supplementary Figure S4A) (49), while the rest of the imino proton peaks were assigned by comparing the spectrum to the previously assigned *Z-G4* spectrum (36). Guanine aromatic proton resonance assignment was obtained by the long-range through-bond coupling method (Supplementary Figure S4B) (50). The solvent exchange analysis revealed that eight out of sixteen guanine imino protons peaks corresponding to G2, G6, G11, G15, G20, G23, G26 and G29 quickly disappeared due to exchange with D_2_O solvent, while the other eight remained intact after 9 days of equilibration in D_2_O (Supplementary Figure S5). This result indicated the formation of a four-layered G4 structure as well as classified the guanines into either outer tetrad layers or inner tetrad layers. Note that the imino proton of G8 was not observable suggesting its involvement in one of the G4 loops.

**Figure 2.**
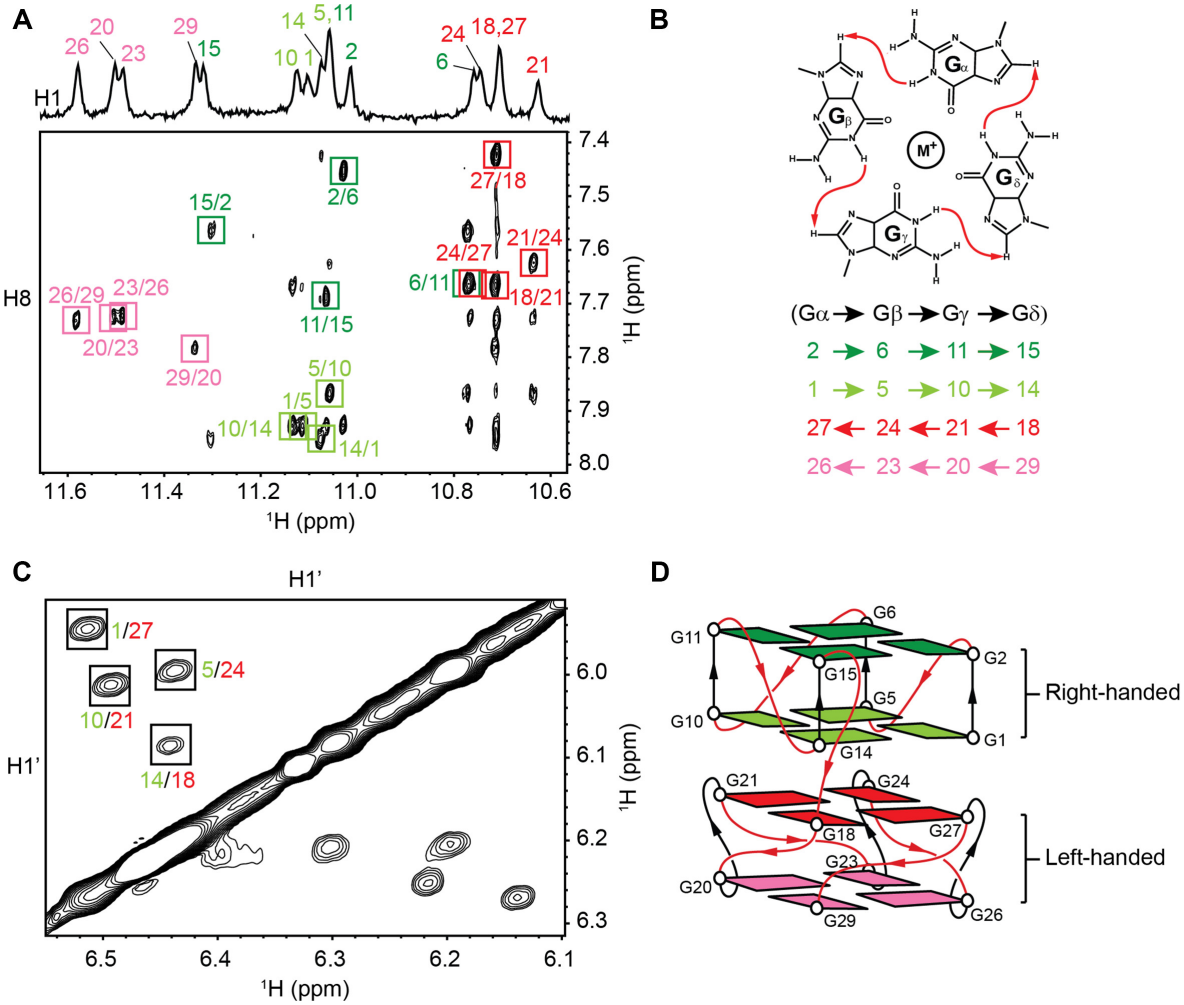
(**A**) NOESY spectrum of *TBA-TT-Block2* (mixing time 200 ms) recorded in the presence of 90 mM K^+^ in H_2_O showing guanine imino (H1)-aromatic (H8) cyclic-connectivity patterns. H1-H8 cross-peaks are labeled with the sequence numbers and shown in distinct colors pertaining to different G-tetrads. (**B**) The schematic of G-tetrad NOE connectivity pattern and cyclic orientations of guanines from each G-tetrads. (**C**) NOESY spectrum of *TBA-TT-Block2* (mixing time 100 ms) showing the strong H1′–H1′ cross-peaks between inner tetrad guanosines. (**D**) Proposed schematic of *TBA-TT-Block2* showing stacking interaction between a right-handed block and a left-handed block. All guanines are color-coded: dark green corresponding to top tetrad, light green for the second tetrad, red for the third tetrad, and pink for the bottom tetrad.

The NOESY spectrum performed at 200 ms mixing time in H_2_O displayed four sets of guanine H1-H8 connectivity patterns in the imino-aromatic proton region (Figure 2A), showing that the G4 core structure was comprised of four G-tetrads, G2•G6•G11•G15, G1•G5•G10•G14, G18•G21•G24•G27 and G20•G23•G26•G29 (Figure 2B). Combining the NOESY data with the solvent exchange results, we deduced that the overall structure can be divided into two blocks of parallel G4, with each block exclusively containing guanines from either the *TBA* sequence or the *Block2* sequence. The two blocks were connected by a TT linker. The NOESY spectrum performed at 300 ms mixing time in D_2_O displayed H8_(*n*)_–H1′_(*n*)_–H8_(*n*+1)_ sequential-walk patterns in the H8–H1′ proton region (Supplementary Figure S6), with missing inter-residue cross-peaks corresponding to residues within some thymine loops. The signal intensities of guanine intra-residue H8–H1′ cross-peaks in the spectrum were uniformly moderate, which implied an all-*anti* conformation in support of the proposed parallel folding topology of the G4 blocks.

The interface between the *TBA* and *Block2* was examined by looking at NOESY cross-peaks between the inner tetrad guanines. The D_2_O NOESY spectrum at 100 ms mixing time (Figure 2C) showed four strong cross-peaks in the H1′–H1′ region corresponding to NOE interactions between G1/G27, G5/G24, G10/G21 and G14/G18. The short spatial distances between the two H1′ protons of the stacking guanosines suggested a 5′-5′ stacking mode between the two sugar groups. Based on this observation, the *TBA* block is deduced to possess a natural backbone progression, while the *Block2*—as previously observed in other left-handed G4s (36,37)—carries a twisted backbone progression, as illustrated in the proposed schematic (Figure 2D).

**Figure 3.**
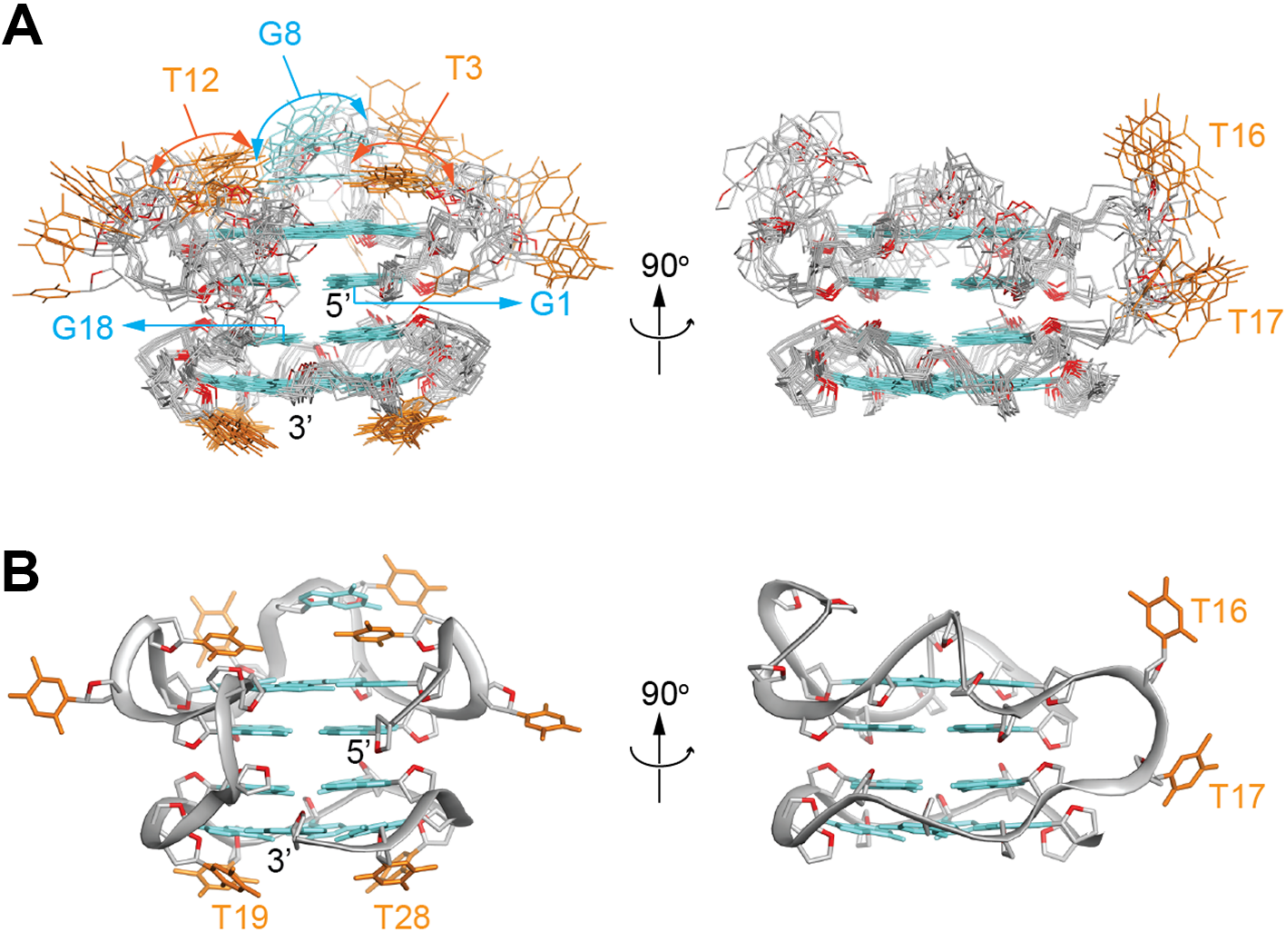
NMR solution structure of *TBA-TT-Block2*. (**A**) Ten superimposed lowest-energy structures. (**B**) Cartoon view of the representative structure. Guanine and thymine bases are shown in cyan and orange respectively. Phosphate backbone is shown in gray; OP1, OP2 and O4′ oxygens are shown in red.

NMR solution structures of *TBA-TT-Block2* were calculated based on distance, angle, hydrogen-bond and planarity constraints obtained from NMR spectral analyses. Ten lowest-energy structures out of the 100 calculated structures were superimposed and presented together with a cartoon representation of the chosen structure (Figure 3). The overall structure indicated the formation of adjacent right- and left-handed G4 blocks, connected by a linker comprising of two thymine residues (T16 and T17). The attached *TBA* sequence adopted a parallel right-handed G4 block, with three connecting loops. One base out of each loop (T3, G8 and T12) was observed to be stacking on top of the 3′ G-tetrad core (G2•G6•G11•G15), while the other bases from the loops (T4, T7, T9 and T13) were found to be projecting out. On the other hand, the attached *Block2* sequence maintained a parallel, left-handed G4 structure, featuring one split-guanine tract (G18/G29). The thymine loops for *Block2* were observed to be stacking on the bottom tetrad layer (G20•G23•G26•G29), as previously observed (36,37) (Supplementary Figure S7). The interface of the two parallel blocks was recognized to comprise of the 5/6-ring stacking mode, with opposing G-tetrad orientations between the two inner tetrads (51). The NMR restraints used as well as the structure calculations statistics are presented in Table 1.

**Table 1.** Statistics of the computed solution structure of *TBA-TT-Block2*

**A. NMR restraints**		
Distance restraints	H_2_O	D_2_O
Intra-residue	8	537
Inter-residue	62	195
Other restraints		
Hydrogen bond	64	
Dihedral angle	16	
Planarity	4	
**B. Structure statistics**		
NOE violations		
Number (>0.2 Å)	0.600 ± 0.699	
Deviations from the ideal geometry		
Bond lengths (Å)	0.003 ± 0.000	
Bond angles (°)	0.681 ± 0.006	
Impropers (°)	0.355 ± 0.004	
Pairwise heavy atom RMSD value (Å)		
G-tetrad core	0.822 ± 0.156	
All heavy atoms	2.359 ± 0.322	

### X-ray crystal structure of *TBA-T-Block2*

The 1D ^1^H NMR and CD spectra of sequences containing both *TBA* and *Block2* with single and double thymine linkers (Supplementary Figure S1) showed that they adopt the same conformation. However, crystals were only obtained for the single thymine linker sequence (*TBA-T-Block2*). We examined the resolved NMR structure of *TBA-TT-Block2* and observed that the linker connects the two G4 blocks, specifically from the first to third G-tetrad (looking from the top). The single thymine of *TBA-T-Block2* was expected to have a similar functionality as its double thymine linker counterpart, preserving the overall structure.

**Figure 4.**
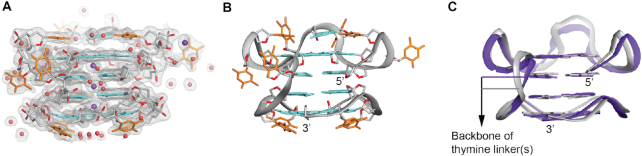
Crystal structure of *TBA-T-Block2*. (**A**) Electron density map (2mF0 – DFc) corresponding to the final refined structure contoured at 1.0σ. (**B**) Cartoon view of the crystal structure of *TBA-T-Block2*, guanine and thymine bases are shown in cyan and orange respectively. Phosphate backbone is shown in gray; OP1, OP2 and O4′ oxygens are shown in red. Potassium ions and water molecules are shown in purple and red respectively. (**C**) Superposition of the NMR solution (gray) and X-ray crystal (purple) structures showing excellent alignment of the backbones and the guanine bases participating in the G-tetrads. Sugars and bases from the loops are removed for clarity. The different thymine linkers are indicated.

After initial processing and model refinement in the P2_1_ space group, strong evidence of crystal twinning appeared (see Materials and Methods). Therefore, diffraction data from two crystals of *TBA-T-Block2* were merged and processed in the P1 space group and the structure was refined using the twin law -*h, k*, -*l* (Table 2). Four molecules were present in the asymmetric unit arranged in two pseudo-equivalent sets, each containing a pair of unimolecular G4s co-axially stacked on each other in a ‘tail to head’ fashion (Supplementary Figure S8A). The T21 and T24 residues from the *Block2* unit of one molecule were stacked on the T3 and T12 residues of the *TBA* unit of the second molecule (Supplementary Figure S8B). The four molecules in the asymmetric unit had an excellent concordance among the guanine cores (pairwise rmsd of 0.347 ± 0.075 Å). However, a rather poor all-atom alignment (pairwise rmsd of 1.306±0.447 Å) was observed due to disorder in the thymine loops, especially in the right-handed *TBA* block. One out of the four molecules was randomly chosen for the purpose of representation of the structure and corresponding electron density (Figure 4A, B).

**Table 2.** Data collection and refinement statistics for the X-ray crystal structure of *TBA-T-Block2*

**Data collection**	
Wavelength (Å)	0.98
Resolution range (Å)	31–1.8
Space group	*P*1
Unit cell	31.12, 39.27, 53.55, 90.036, 93.029, 89.946
Unique reflections	22 501 (3070)
Multiplicity	4.1
Completeness (%)	95.8 (88)
Mean *I*/sigma(*I*)	10.5 (5.8)
Wilson *B*-factor (Å^2^)	25.3
*R*-sym	0.115 (0.359)
CC1/2	0.99 (0.82)
**Refinement**	
Reflections used in refinement	22 497 (2366)
Reflections used for *R*-free	1131 (121)
*R*-work	0.164 (0.263)
*R*-free	0.202 (0.265)
Refined twinning fraction	0.49 for -*h*, *k*, -*l*
Number of non-hydrogen atoms	2545
Macromolecules	2364
Potassium ions	15
Water	166
DNA residues	112
RMS bond lengths (Å)	0.017
RMS bond angles (°)	1.36
Average *B*-factor	29.9
Macromolecules	30.2
Potassium ions	17.6
Water	26.4

Statistics for the highest-resolution shell (1.9–1.8 Å) are shown in parentheses.

The crystal structure showed a high degree of similarity with the solution NMR structure presented above (pairwise rmsd of 1.62 Å, excluding residues T4, T7, T13 and T17). The backbone and the guanine bases involved in the G-tetrad had an excellent overlap between the two structures (Figure 4C). For the left-handed *Block2*, the structure consisted of single thymine loops (T19, T22, T25 and T28) connecting the G-tracts, which were observed to be nicely overlapped (Supplementary Figure S9A). For the right-handed *TBA* block, one base (T3, G8 and T12) in each di- or tri-nucleotide loops was stacked upon the G-tetrad, whereas the remaining one(s) projected out of the tetrad. The capping bases were found to be superimposed well, while the protruding bases were not (Supplementary Figure S9B). This is not surprising as the electron density did not fit convincingly around these protruding thymine bases. In addition, our NMR data did not show any inter-residue NOE cross-peaks for T4 and T13, while T7 had cross-peaks with only G8. Both the techniques suggested that these three thymine residues were disordered. Overall, the high degree of agreement between the crystal structure of *TBA-T-Block2* and the solution NMR structure of *TBA-TT-Block2* indicated that the two sequences folded into similar structures regardless of the linker length, and thus confirmed the formation of a structure with adjacent right- and left-handed G-quadruplexes.

### Structural analysis

#### Parallel backbone progression

**Figure 5.**
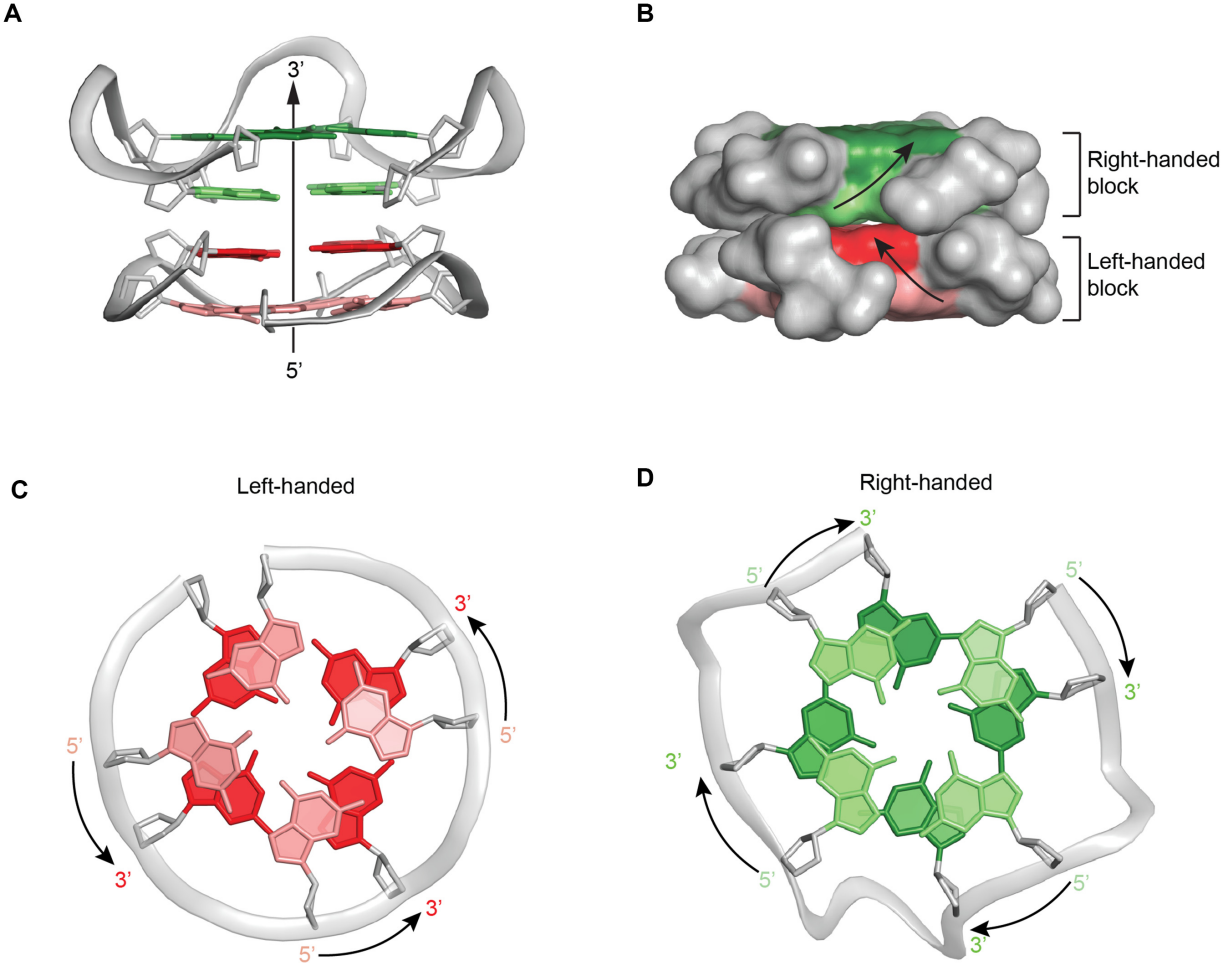
Backbone orientation and stacking of the right- and left-handed blocks in *TBA-T-Block2*. (**A**) Cartoon view showing overall backbone orientation of two blocks in *TBA-T-Block2*. (**B**) Surface view showing the zig-zag direction of the sugar-phosphate backbone in the right and left-handed blocks. (**C**) View from 5′-end of the left-handed block showing anti-clockwise backbone progression from 5′-3′ guanine bases. (**D**) View from 5′-end of the right-handed block showing clockwise backbone progression from 5′-3′ guanine bases.

The core structure of both *TBA-TT-Block2* and *TBA-T-Block2* adopt the same topology which includes four parallel strands, with the right-handed block (*TBA*) exposing the 3′ G-tetrad and the left-handed block (*Block2*) exposing the 5′ G-tetrad towards the solvent (Figure 5A). However, unlike the general parallel strands of a right-handed G4, the sugar-phosphate backbone of each strand in our structure followed a zig-zag orientation as illustrated in Figure 5B.

#### 5′-5′ sugar stacking interface between the left- and right G4 blocks

The backbone progression of right-handed and left-handed G4s are one of the defining features that differentiate them from each other. To visualize the difference, we considered a vector from the C5′ atom towards the C3′ atom in one sugar group to determine the local orientation of the sugar, and we regarded the vector from the 5′ G-tetrad to 3′ G-tetrad as an indicator of the backbone progression orientation (Supplementary Figure S10A). In a right-handed GG step, these two vectors are less than 90° apart, while in a left-handed GG step they are more than 90° apart from each other (Supplementary Figure S10B). These features allowed the formation of a 5′–5′ sugar stacking mode while maintaining the overall 5′–3′ backbone progression in the left- to right-handed G4 junction as observed in the NMR and crystal structures obtained in this study (Supplementary Figure S10C).

#### Opposite-polarity G-tetrad stacking

Another distinguishing feature between right- and left-handed G-tetrads are their cyclic orientations (or base alignment H-bond directionality) relative to the backbone. Viewing from the 5′ side, the cyclic orientation is clockwise in a parallel right-handed G4 structure (Figure 5D) and anti-clockwise in a parallel left-handed counterpart (Figure 5C). Hence, instead of the same-polarity G-tetrad stacking observed for a parallel structure, an opposite-polarity G-tetrad stacking was detected between the right-handed and left-handed parallel G4 blocks.

#### Backbone dihedrals

Backbone dihedrals are yet another contrasting feature between the left- and right-handed G4s. We calculated the torsion angles for the dinucleotide steps, G19–G20 and G22–G23 (G25–G26 step was excluded for its divergent values) in the left-handed block as well as dinucleotide steps G1–G2, G5–G6, G10–G11 and G14–G15 in the right-handed block. Measured values are listed in Supplementary Table S1, which clearly depicts differences in the angle values. Some of the dihedral angles, namely ϵ and ζ, had more striking differences between the two G4 blocks than the other angles. We further observed that both the left-handed block and right-handed block had slightly deviated backbone torsional angle values compared to the regular left-handed (*Z-G4*) and right-handed G4s respectively (36,37), potentially in order to accommodate the hybridization.

### Single-nucleotide loops are preferred or required for the left-handed G-quadruplex formation

The solved crystal structure of *Z-G4* (PDB ID: 4U5M) (36) was shown to adopt a left-handed G4 despite the original right-handed conformation of the isolated *Block1* sequence 5′-d(TGGTGGTGGTGGTT)-3′ (37) (Supplementary Figure S11A). In both *Z-G4* and our *TBA*-*T*-*Block2* sequences, *Block2* motifs were shown to drive a different folding topology on the attached sequences (*Block1* and *TBA*, respectively). However, the *Block1* sequence was converted into a parallel left-handed structure, while the *TBA* sequence was changed into a parallel right-handed structure (Supplementary Figure S11B). Examining the differences between the attached sequences, we noticed that while *Block1* consisted of three single-T loops, *TBA* comprised of relatively longer loops (two TT loops and one TGT loop).

To understand the effect of the loop composition in the attached sequences, we decided to perform a spectroscopic study on a *Z-G4* variance, *Z-G4-T4mod*, introducing one additional thymine in the first loop of *Block1* (Supplementary Figures S12 and S13A). A single unambiguous spectral assignment based on ^15^N site-specific labelling and D_2_O exchange experiments (Supplementary Figure S12) indicated that the first guanine (G2) of *Z-G4-T4mod* is an inner tetrad residue, similar to that of *TBA-TT-Block2* sequence, and opposite of that of *Z-G4*. Furthermore, the 1D ^1^H NMR spectrum of *Z-G4-T4mod* showed a distribution of imino protons which is also similar to that of *TBA-TT-Block2*, with outer and inner imino protons not clearly separated (Supplementary Figure S12 and S13B), as opposed to a clear separation in the case of *Z-G4*. The CD spectra of all the three sequences (*Z-G4*,*Z-G4-T4mod* and *TBA-TT-Block2*) revealed a peak at ∼245 nm and a trough at ∼270–275 nm, indicating the presence of left-handed structures (Supplementary Figure S13C). In addition, we probed another characteristic of left-handed G4, which is the observation of the NOESY cross-peaks of guanine imino to amino protons (52). Both amino protons of the outer guanines from a left-handed G4 block are often hydrogen bonded (Supplementary Figure S13D) (36), resulting in sharp NOE cross-peaks with the neighbouring imino protons. The 200–300 ms NOESY spectra of the three sequences (*Z-G4*,*Z-G4-T4mod* and *TBA-TT-Block2*) displayed five, three and three sharp imino-amino NOE cross-peaks respectively (Supplementary Figure S13E), most likely corresponding to the two left-handed blocks in *Z-G4*, and a single left-handed block in *Z-G4-T4mod* and *TBA-TT-Block2*. Combining all the results, we proposed that the *Z-G4-T4mod* sequence adopts a right- and left-handed G4 hybrid folding topology, similar to that of *TBA-TT-Block2*. Thus, an increase of the size of a single-residue loop from T to TT switched the *Block1* from a left-handed G4 conformation in *Z-G4* to a right-handed G4 in *Z-G4-T4mod*. This result suggests that single-residue loops are preferred or even required for the formation of a two-layer left-handed G4 block. This was supported by NMR experiments from a previous study (37), where substitutions of a T loop with a TT loop (rather than a C or A loop) in a single block of the *2xBlock2* sequence produced similar results.

### Comparison with other helical junctions

There are various kinds of junctions between two types of helical structure of DNA, including compact junctions—where all nucleobases from the two types of structures stay in a stacking base-pairing interaction, and open junctions—where there exists a break in the overall structure consisting of one or more non base-pairing residue/s (Supplementary Figure S14). For instance, a four-way junction (or Holliday junction) (53) has been shown to accommodate all the bases in pairing conformations, resulting in a compact structure mediated by strong van der Walls, hydrogen bonding and stacking interactions between the four participating oligonucleotide strands (54). On the other hand, B-to-Z DNA junctions require extruding bases, one from each DNA strands, resulting in more relaxed backbone configuration between the right- and left-handed DNA helices (5). The junction between quadruplex and duplex has been studied in detail via G4-hairpin structures (55). Two different orientations of quadruplex–duplex junctions (coaxial and orthogonal) have been identified. Structures of different G4-hairpin with coaxial junctions were reported, some accommodated the direct stacking between the G-tetrad and the Watson–Crick base pair from the hairpin, while the other requires some base-pairing mismatch (A•G) in between the two structures due to the considerable size difference. In contrary, the structures of G4-hairpin with orthogonal junction revealed a loose junction, where there exists multiple non base-pairing residues between the double and quadruple helices. Here, the left- to right-handed G4 junction is shown to simply consist of stacking interaction between two G-tetrads of different cyclic orientations, much like the interaction of right-handed parallel G4 dimer (30,56).

## CONCLUSION

The first structure of a right- and left-handed hybrid G-quadruplex has been solved via both NMR spectroscopy and X-ray crystallography. The original right-handed, anti-parallel thrombin-binding aptamer G-quadruplex is converted into a right-handed, parallel G-quadruplex when connected with the *Block2* sequence, which is a left-handed G4 forming motif. The final hybrid structure comprises of two parallel G-quadruplex blocks with different helical orientations, connected by a linker. Our structural analyses indicate that the structure retains the individual features of both right- and left-handed G-quadruplexes.

## DATA AVAILABILITY

The coordinates for the NMR solution and X-ray crystal structures have been deposited in the Protein Data Bank (PDB ID: 6JCE and 6QJO).

## Supplementary Material

gkz349_Supplemental_File

## References

[1] Wing R., Drew H., Takano T., Broka C., Tanaka S., Itakura K., Dickerson R.E. Crystal structure analysis of a complete turn of B-DNA. Nature. 1980; 287:755–758. 7432492 10.1038/287755a0

[2] Wang A.H., Quigley G.J., Kolpak F.J., Crawford J.L., van Boom J.H., van der Marel G., Rich A. Molecular structure of a left-handed double helical DNA fragment at atomic resolution. Nature. 1979; 282:680–686. 514347 10.1038/282680a0

[3] Liu R., Liu H., Chen X., Kirby M., Brown P.O., Zhao K. Regulation of CSF1 promoter by the SWI/SNF-like BAF complex. Cell. 2001; 106:309–318. 11509180 10.1016/s0092-8674(01)00446-9

[4] Oh D.B., Kim Y.G., Rich A. Z-DNA-binding proteins can act as potent effectors of gene expression in vivo. Proc. Natl. Acad. Sci. U.S.A. 2002; 99:16666–16671. 12486233 10.1073/pnas.262672699 PMC139201

[5] Ha S.C., Lowenhaupt K., Rich A., Kim Y.G., Kim K.K. Crystal structure of a junction between B-DNA and Z-DNA reveals two extruded bases. Nature. 2005; 437:1183–1186. 16237447 10.1038/nature04088

[6] Rich A., Zhang S. Timeline: Z-DNA: the long road to biological function. Nat. Rev. Genet. 2003; 4:566–572. 12838348 10.1038/nrg1115

[7] Schwartz T., Rould M.A., Lowenhaupt K., Herbert A., Rich A. Crystal structure of the Zalpha domain of the human editing enzyme ADAR1 bound to left-handed Z-DNA. Science. 1999; 284:1841–1845. 10364558 10.1126/science.284.5421.1841

[8] Rothenburg S., Deigendesch N., Dittmar K., Koch-Nolte F., Haag F., Lowenhaupt K., Rich A. A PKR-like eukaryotic initiation factor 2alpha kinase from zebrafish contains Z-DNA binding domains instead of dsRNA binding domains. Proc. Natl. Acad. Sci. U.S.A. 2005; 102:1602–1607. 15659550 10.1073/pnas.0408714102 PMC547857

[9] Fry M. Tetraplex DNA and its interacting proteins. Front. Biosci. 2007; 12:4336–4351. 17485378 10.2741/2391

[10] Monchaud D., Teulade-Fichou M.P. A hitchhiker's guide to G-quadruplex ligands. Org. Biomol. Chem. 2008; 6:627–636. 18264563 10.1039/b714772b

[11] Fu B., Huang J., Chen Y., Wang Y., Xue T., Xu G., Wang S., Zhou X. Right-handed and left-handed G-quadruplexes have the same DNA sequence: distinct conformations induced by an organic small molecule and potassium. Chem. Commun. (Camb.). 2016; 52:10052–10055. 27452654 10.1039/c6cc04866h

[12] Zhao C., Song H., Scott P., Zhao A., Tateishi-Karimata H., Sugimoto N., Ren J., Qu X. Mirror-image dependence: targeting enantiomeric G-quadruplex DNA using triplex metallohelices. Angew. Chem. Int. Ed. Engl. 2018; 57:15723–15727. 30311333 10.1002/anie.201809207

[13] Gellert M., Lipsett M.N., Davies D.R. Helix formation by guanylic acid. Proc. Natl. Acad. Sci. U.S.A. 1962; 48:2013–2018. 13947099 10.1073/pnas.48.12.2013 PMC221115

[14] Sen D., Gilbert W. Formation of parallel four-stranded complexes by guanine-rich motifs in DNA and its implications for meiosis. Nature. 1988; 334:364–366. 3393228 10.1038/334364a0

[15] Smith F.W., Feigon J. Quadruplex structure of Oxytricha telomeric DNA oligonucleotides. Nature. 1992; 356:164. 1545871 10.1038/356164a0

[16] Wang Y., Patel D.J. Solution structure of the human telomeric repeat d[AG3(T2AG3)3] G-tetraplex. Structure. 1993; 1:263–282. 8081740 10.1016/0969-2126(93)90015-9

[17] Phillips K., Dauter Z., Murchie A.I., Lilley D.M., Luisi B. The crystal structure of a parallel-stranded guanine tetraplex at 0.95 Å resolution. J. Mol. Biol. 1997; 273:171–182. 9367755 10.1006/jmbi.1997.1292

[18] Parkinson G.N., Lee M.P., Neidle S. Crystal structure of parallel quadruplexes from human telomeric DNA. Nature. 2002; 417:876–880. 12050675 10.1038/nature755

[19] Haider S., Parkinson G.N., Neidle S. Crystal structure of the potassium form of an Oxytricha nova G-quadruplex. J. Mol. Biol. 2002; 320:189–200. 12079378 10.1016/S0022-2836(02)00428-X

[20] Crnugelj M., Sket P., Plavec J. Small change in a G-rich sequence, a dramatic change in topology: new dimeric G-quadruplex folding motif with unique loop orientations. J. Am. Chem. Soc. 2003; 125:7866–7871. 12823005 10.1021/ja0348694

[21] Davis J.T. G-quartets 40 years later: from 5′-GMP to molecular biology and supramolecular chemistry. Angew. Chem. Int. Ed. Engl. 2004; 43:668–698. 14755695 10.1002/anie.200300589

[22] Luu K.N., Phan A.T., Kuryavyi V., Lacroix L., Patel D.J. Structure of the human telomere in K+ solution: an intramolecular (3 + 1) G-quadruplex scaffold. J. Am. Chem. Soc. 2006; 128:9963–9970. 16866556 10.1021/ja062791w PMC4692383

[23] Burge S., Parkinson G.N., Hazel P., Todd A.K., Neidle S. Quadruplex DNA: sequence, topology and structure. Nucleic Acids Res. 2006; 34:5402–5415. 17012276 10.1093/nar/gkl655 PMC1636468

[24] Dai J., Dexheimer T.S., Chen D., Carver M., Ambrus A., Jones R.A., Yang D. An intramolecular G-quadruplex structure with mixed parallel/antiparallel G-strands formed in the human BCL-2 promoter region in solution. J. Am. Chem. Soc. 2006; 128:1096–1098. 16433524 10.1021/ja055636a PMC2556172

[25] Patel D.J., Phan A.T., Kuryavyi V. Human telomere, oncogenic promoter and 5′-UTR G-quadruplexes: diverse higher order DNA and RNA targets for cancer therapeutics. Nucleic Acids Res. 2007; 35:7429–7455. 17913750 10.1093/nar/gkm711 PMC2190718

[26] Lim K.W., Amrane S., Bouaziz S., Xu W., Mu Y., Patel D.J., Luu K.N., Phan A.T. Structure of the human telomere in K+ solution: a stable basket-type G-quadruplex with only two G-tetrad layers. J. Am. Chem. Soc. 2009; 131:4301–4309. 19271707 10.1021/ja807503g PMC2662591

[27] Webba da Silva M., Trajkovski M., Sannohe Y., Ma’ani Hessari N., Sugiyama H., Plavec J. Design of a G‐quadruplex topology through glycosidic bond angles. Angew. Chem. Int. Ed. Engl. 2009; 48:9167–9170. 19882602 10.1002/anie.200902454

[28] Kuryavyi V., Phan A.T., Patel D.J. Solution structures of all parallel-stranded monomeric and dimeric G-quadruplex scaffolds of the human c-kit2 promoter. Nucleic Acids Res. 2010; 38:6757–6773. 20566478 10.1093/nar/gkq558 PMC2965254

[29] Russo Krauss I., Spiridonova V., Pica A., Napolitano V., Sica F. Different duplex/quadruplex junctions determine the properties of anti-thrombin aptamers with mixed folding. Nucleic Acids Res. 2015; 44:983–991. 26673709 10.1093/nar/gkv1384 PMC4737158

[30] Do N.Q., Chung W.J., Truong T.H.A., Heddi B., Phan A.T. G-quadruplex structure of an anti-proliferative DNA sequence. Nucleic Acids Res. 2017; 45:7487–7493. 28549181 10.1093/nar/gkx274 PMC5499593

[31] Greco M.L., Kotar A., Rigo R., Cristofari C., Plavec J., Sissi C. Coexistence of two main folded G-quadruplexes within a single G-rich domain in the EGFR promoter. Nucleic Acids Res. 2017; 45:10132–10142. 28973461 10.1093/nar/gkx678 PMC5737278

[32] Dvorkin S.A., Karsisiotis A.I., Webba da Silva M. Encoding canonical DNA quadruplex structure. Sci. Adv. 2018; 4:eaat3007. 30182059 10.1126/sciadv.aat3007 PMC6118410

[33] Wan C., Fu W., Jing H., Zhang N. NMR solution structure of an asymmetric intermolecular leaped V-shape G-quadruplex: selective recognition of the d (G2NG3NG4) sequence motif by a short linear G-rich DNA probe. Nucleic Acids Res. 2018; 47:1544–1556. 10.1093/nar/gky1167 PMC6379650 30445650

[34] Sengar A., Vandana J.J., Chambers V.S., Di Antonio M., Winnerdy F.R., Balasubramanian S., Phan A.T. Structure of a (3+1) hybrid G-quadruplex in the *PARP1* promoter. Nucleic Acids Res. 2019; 47:1564–1572. 30551210 10.1093/nar/gky1179 PMC6379715

[35] Mergny J.L., Sen D. DNA Quadruple helices in nanotechnology. Chem. Rev. 2019; 119:6290–6325. 30605316 10.1021/acs.chemrev.8b00629

[36] Chung W.J., Heddi B., Schmitt E., Lim K.W., Mechulam Y., Phan A.T. Structure of a left-handed DNA G-quadruplex. Proc. Natl. Acad. Sci. U.S.A. 2015; 112:2729–2733. 25695967 10.1073/pnas.1418718112 PMC4352798

[37] Bakalar B., Heddi B., Schmitt E., Mechulam Y., Phan A.T. A minimal sequence for left-handed G-quadruplex formation. Angew. Chem. Int. Ed. Engl. 2019; 58:2331–2335. 30481397 10.1002/anie.201812628

[38] Macaya R.F., Schultze P., Smith F.W., Roe J.A., Feigon J. Thrombin-binding DNA aptamer forms a unimolecular quadruplex structure in solution. Proc. Natl. Acad. Sci. U.S.A. 1993; 90:3745–3749. 8475124 10.1073/pnas.90.8.3745 PMC46378

[39] Kretz C.A., Stafford A.R., Fredenburgh J.C., Weitz J.I. HD1, a thrombin-directed aptamer, binds exosite 1 on prothrombin with high affinity and inhibits its activation by prothrombinase. J. Biol. Chem. 2015; 290:4813. 25713405 10.1074/jbc.A114.607359 PMC4335218

[40] Lee W., Tonelli M., Markley J.L. NMRFAM-SPARKY: enhanced software for biomolecular NMR spectroscopy. Bioinformatics. 2015; 31:1325–1327. 25505092 10.1093/bioinformatics/btu830 PMC4393527

[41] Schwieters C.D., Kuszewski J.J., Tjandra N., Clore G.M. The Xplor-NIH NMR molecular structure determination package. J. Magn. Reson. 2003; 160:65–73. 12565051 10.1016/s1090-7807(02)00014-9

[42] Kabsch W. Xds. Acta Crystallogr. D Biol. Crystallogr. 2010; 66:125–132. 20124692 10.1107/S0907444909047337 PMC2815665

[43] Afonine P.V., Grosse-Kunstleve R.W., Echols N., Headd J.J., Moriarty N.W., Mustyakimov M., Terwilliger T.C., Urzhumtsev A., Zwart P.H., Adams P.D. Towards automated crystallographic structure refinement with phenix.refine. Acta Crystallogr. D Biol. Crystallogr. 2012; 68:352–367. 22505256 10.1107/S0907444912001308 PMC3322595

[44] Headd J.J., Echols N., Afonine P.V., Grosse-Kunstleve R.W., Chen V.B., Moriarty N.W., Richardson D.C., Richardson J.S., Adams P.D. Use of knowledge-based restraints in phenix.refine to improve macromolecular refinement at low resolution. Acta Crystallogr. D Biol. Crystallogr. 2012; 68:381–390. 22505258 10.1107/S0907444911047834 PMC3322597

[45] Emsley P., Lohkamp B., Scott W.G., Cowtan K. Features and development of Coot. Acta Crystallogr. D Biol. Crystallogr. 2010; 66:486–501. 20383002 10.1107/S0907444910007493 PMC2852313

[46] Karsisiotis A.I., Hessari N.M., Novellino E., Spada G.P., Randazzo A., Webba da Silva M. Topological characterization of nucleic acid G-quadruplexes by UV absorption and circular dichroism. Angew. Chem. Int. Ed. Engl. 2011; 50:10645–10648. 21928459 10.1002/anie.201105193

[47] Vorlickova M., Kejnovska I., Sagi J., Renciuk D., Bednarova K., Motlova J., Kypr J. Circular dichroism and guanine quadruplexes. Methods. 2012; 57:64–75. 22450044 10.1016/j.ymeth.2012.03.011

[48] Del Villar-Guerra R., Trent J.O., Chaires J.B. G-Quadruplex Secondary Structure Obtained from Circular Dichroism Spectroscopy. Angew. Chem. Int. Ed. Engl. 2018; 57:7171–7175. 29076232 10.1002/anie.201709184 PMC5920796

[49] Phan A.T., Patel D.J. A site-specific low-enrichment (15)N,(13)C isotope-labeling approach to unambiguous NMR spectral assignments in nucleic acids. J. Am. Chem. Soc. 2002; 124:1160–1161. 11841271 10.1021/ja011977m

[50] Phan A.T. Long-range imino proton-13C J-couplings and the through-bond correlation of imino and non-exchangeable protons in unlabeled DNA. J. Biomol. NMR. 2000; 16:175–178. 10723997 10.1023/a:1008355231085

[51] Lech C.J., Phan A.T., Michel-Beyerle M.E., Voityuk A.A. Influence of base stacking geometry on the nature of excited states in G-quadruplexes: a time-dependent DFT study. J. Phys. Chem. B. 2015; 119:3697–3705. 25654765 10.1021/jp512767j

[52] Adrian M., Winnerdy F.R., Heddi B., Phan A.T. Rotation of Guanine Amino Groups in G-Quadruplexes: A Probe for Local Structure and Ligand Binding. Biophys J. 2017; 113:775–784. 28834714 10.1016/j.bpj.2017.05.053 PMC5567425

[53] Holliday R. A mechanism for gene conversion in fungi. Genet. Res. 1964; 5:282–304. 10.1017/S0016672308009476 18976517

[54] Ortiz-Lombardia M., Gonzalez A., Eritja R., Aymami J., Azorin F., Coll M. Crystal structure of a DNA Holliday junction. Nat. Struct. Biol. 1999; 6:913–917. 10504723 10.1038/13277

[55] Lim K.W., Phan A.T. Structural basis of DNA quadruplex-duplex junction formation. Angew. Chem. Int. Ed. Engl. 2013; 52:8566–8569. 23794476 10.1002/anie.201302995

[56] Do N.Q., Phan A.T. Monomer-dimer equilibrium for the 5′-5′ stacking of propeller-type parallel-stranded G-quadruplexes: NMR structural study. Chemistry. 2012; 18:14752–14759. 23019076 10.1002/chem.201103295

