# Supplementary material for: NMR solution and X-ray crystal structures of a DNA molecule containing both right- and left-handed parallel-stranded G-quadruplexes
Source: Nucleic Acids Res. 2019 Jun 19;47(15):8272–81. doi: 10.1093/nar/gkz349 (PMC6735952; doi:10.1093/nar/gkz349)
Supplement: gkz349_Supplemental_File [file gkz349_supplemental_file.pdf]

NMR solution and X-ray crystal structures of a DNA molecule containing both right- and left-handed parallel-stranded G-quadruplexes

Fernaldo Richtia Winnerdy[1], Blaž Bakalar[1], Arijit Maity[1], J Jeya Vandana[1], Yves Mechulam[2], Emmanuelle Schmitt[2] and Anh Tuân Phan[1,*]

[1] School of Physical and Mathematical Sciences, Nanyang Technological University, Singapore 637371, Singapore

[2] Laboratoire de Biochimie, UMR 7654, CNRS, Ecole Polytechnique, Palaiseau 91128, France

* To whom correspondence should be addressed. Email: phantuan@ntu.edu.sg

SUPPORTING INFORMATION

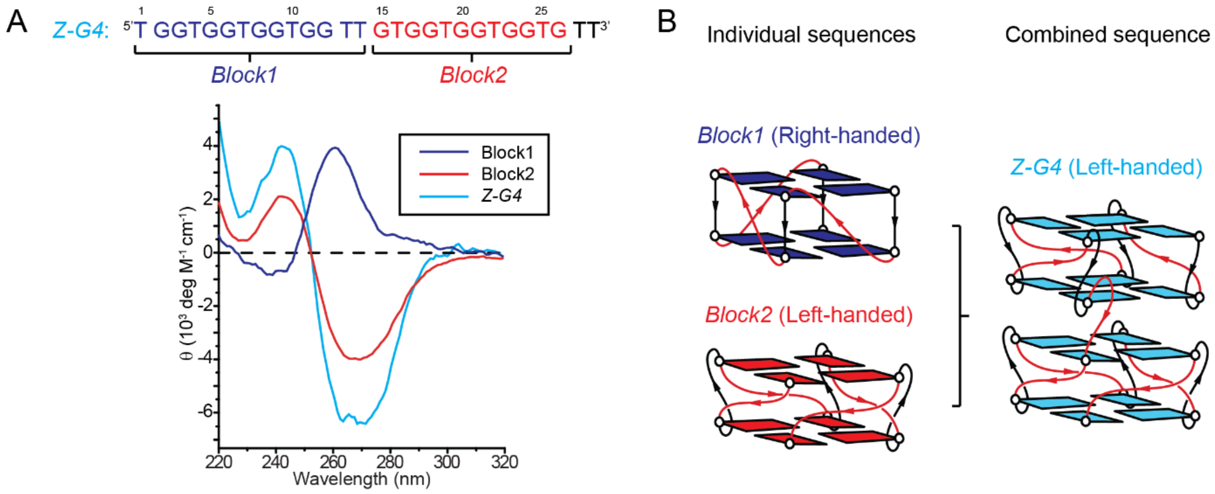

Figure S1. (A) Sequences and CD spectra of *Block1*, *Block2* and *Z-G4*. The CD data indicate the conversion of the initial right-handed conformation of *Block1* towards left-handed conformation when attached to *Block2*. CD spectrum of *Z-G4* was measured with a longer incubation time after annealing than in (1) to reduce minor conformations, hence the slightly different spectrum. (B) Schematics of G4s for individual sequences of *Block1* and *Block2* as well as the combined sequence (*Z-G4*).

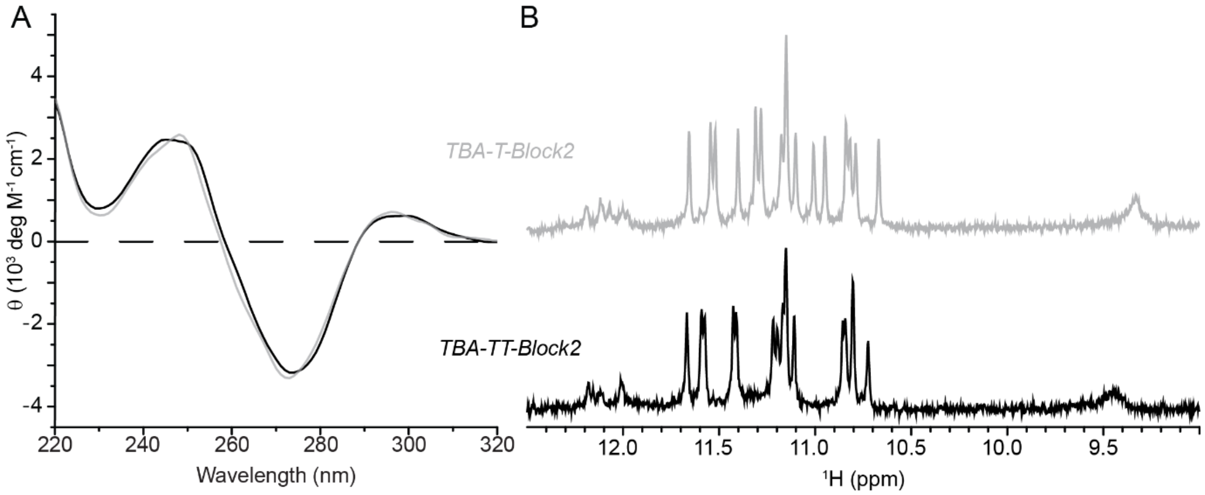

Figure S2. Comparison of (A) CD and (B) 1D ^{1}H NMR spectra of *TBA-TT-Block2* (black) and *TBA-T-Block2* (grey). The length of the linker does not significantly impact the spectral characteristics of the two constructs.

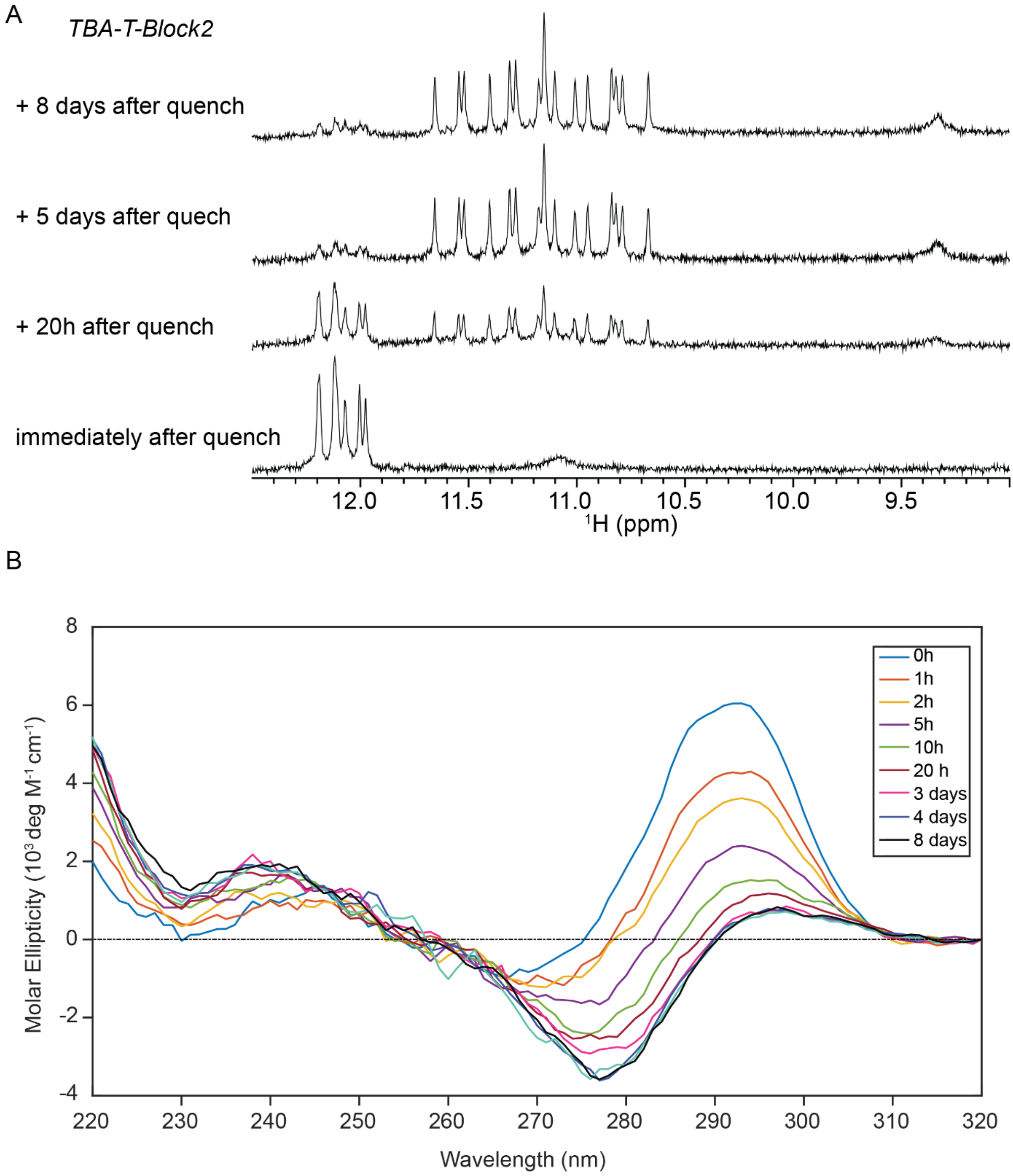

Figure S3. Time-series 1D ^{1}H NMR and CD spectra measurements of *TBA-T-Block2*. (A) NMR and (B) CD spectra of *TBA-T-Block2* measured in 100 μM concentration showing the disappearance of the original *TBA* fold and emergence of the fully folded *TBA-T-Block2* with time.

A

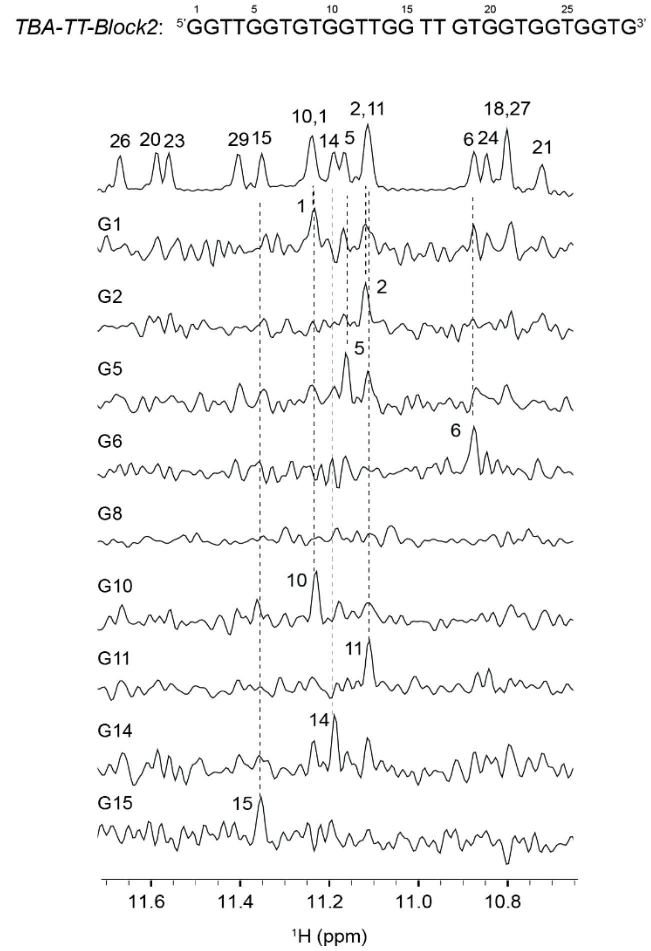

B

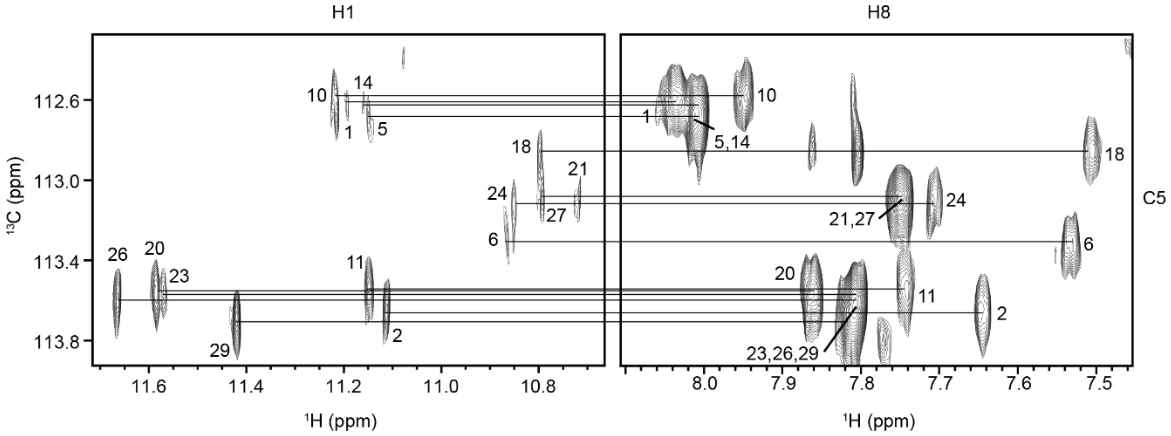

Figure S4. Unambiguous resonance assignments of *TBA-TT-Block2*. (A) Sequence of *TBA-TT-Block2* and assignment of its guanine imino protons by site specific ^{15}N-labeling method (2). (B) Assignment of guanine aromatic protons from the long-range through-bond coupling method (3).

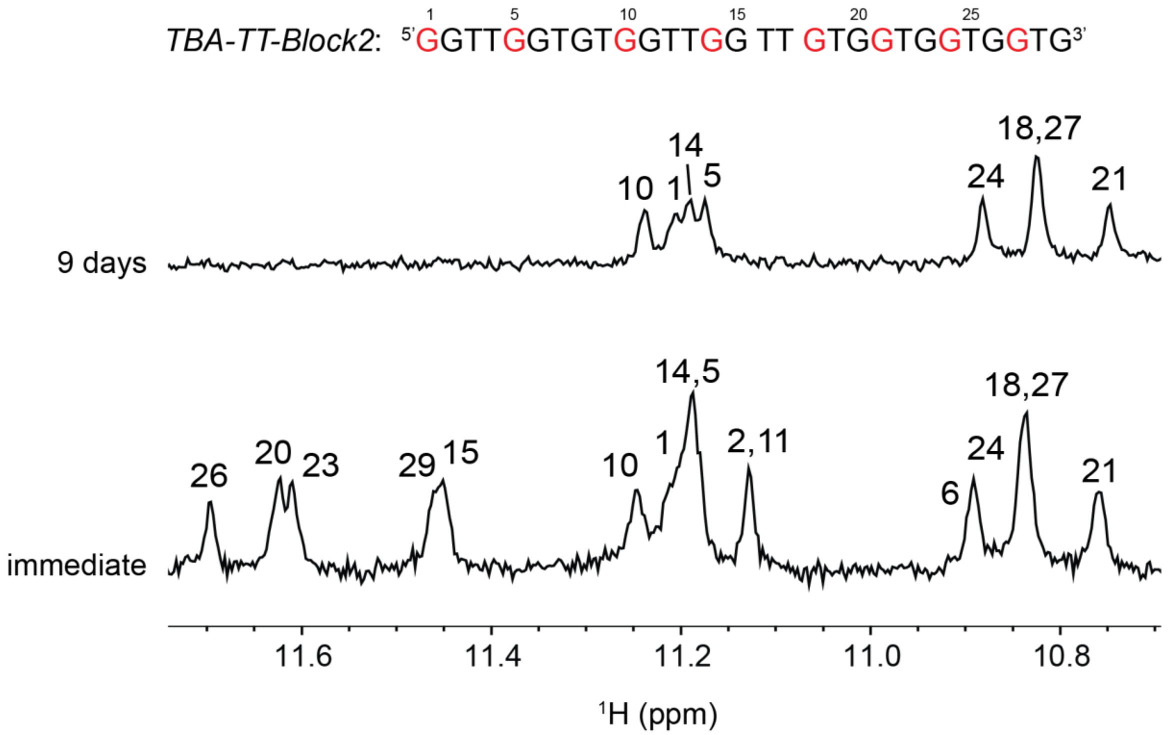

Figure S5. Solvent exchange of the *TBA-TT-Block2* sample at 25°C. The sequence of *TBA-TT-Block2* is displayed with the guanines containing protected imino protons colored red. The bottom spectrum was recorded immediately after adding 100% D_2O with the corresponding imino protons assignments shown. The top spectrum was recorded 9 days after addition of D_2O, showing disappearance of outer tetrad imino proton peaks.

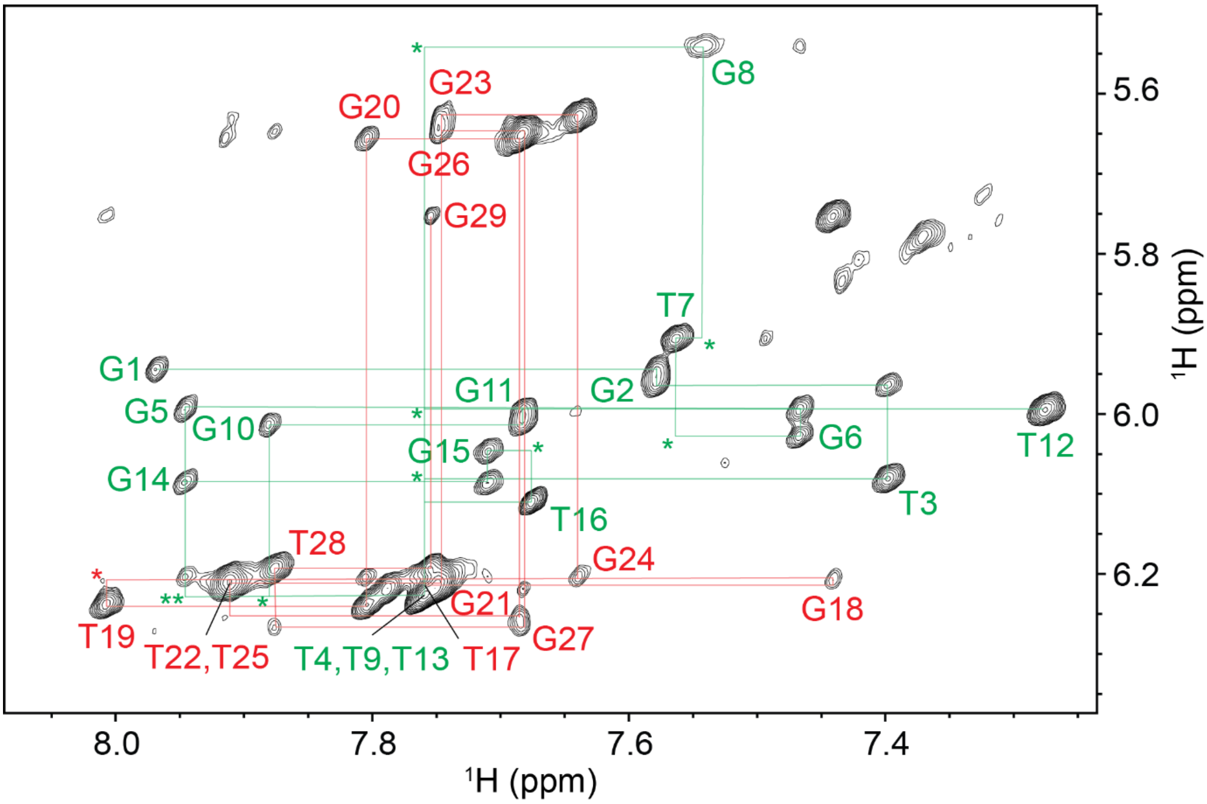

Figure S6. D_2O NOESY spectrum (mixing time 300 ms) of *TBA-TT-Block2* at 25°C. Sequential $H8_{(n)}$-$H1'_{(n)}$-$H8_{(n+1)}$ walk of the right-handed and left-handed blocks are shown in green and red respectively. Intra residue H8-H1' cross-peaks are marked with the corresponding residue number. Missing inter-residue cross-peaks are represented with *.

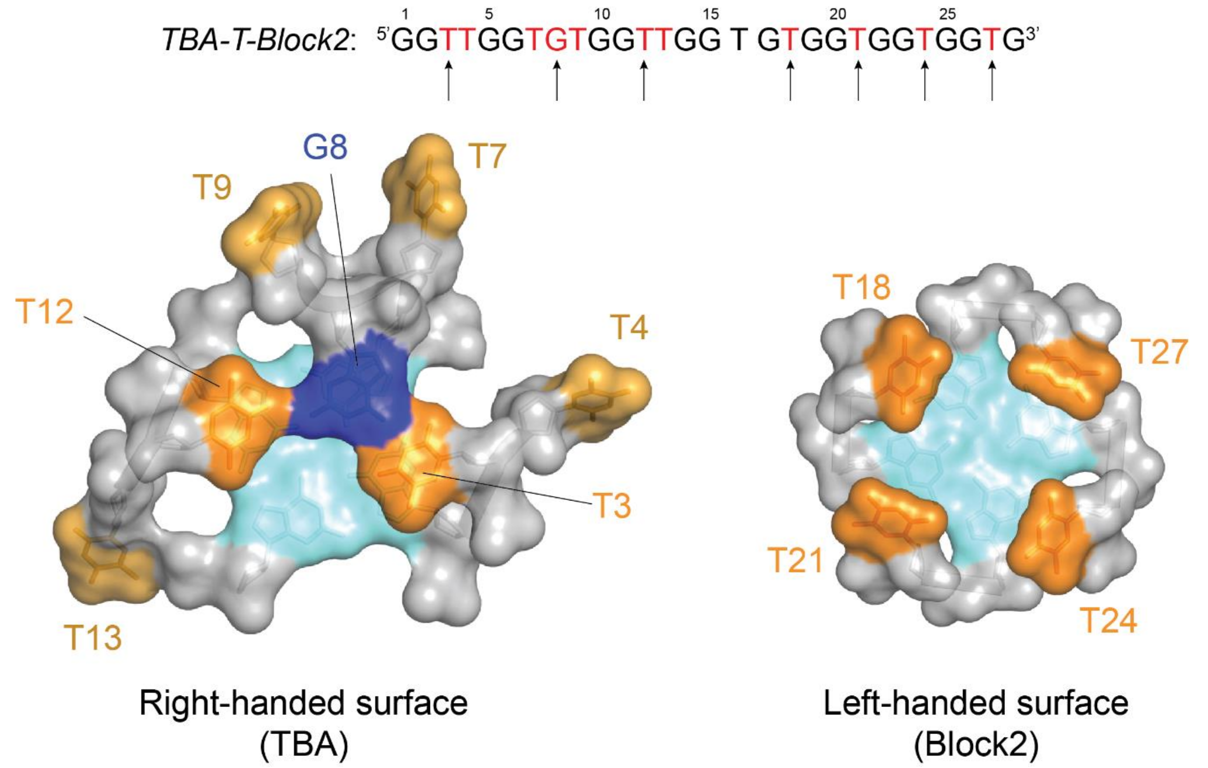

Figure S7. Surface representations of the capping residues of the two blocks show different capping modes between the right- and left-handed blocks. The sequence of *TBA-T-Block2* is shown, the loops are colored red, and the capping bases are marked with arrows. One base (T3, G8 and T12) of each loop of the *TBA* block is shown to be capping the G-tetrad, while other bases are shown projecting out. All the bases of the thymine loops of the *Block2* are shown capping the G-tetrad, consistent with previous studies (1,4).

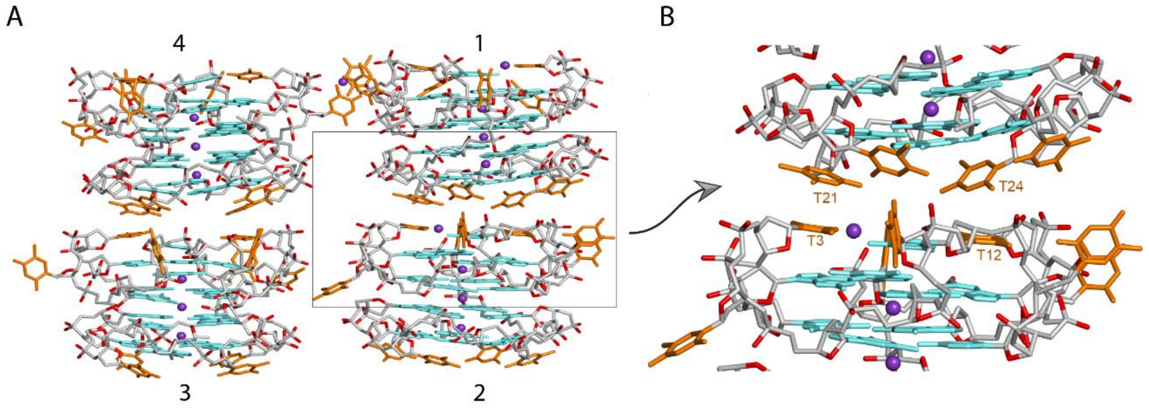

Figure S8. (A) Contents of the asymmetric unit of *TBA-T-Block2* crystal structures. Subunits are numbered in numerical order. (B) Zoomed-in portion of panel A showing stacking between thymine residues of co-axially stacked quadruplex units.

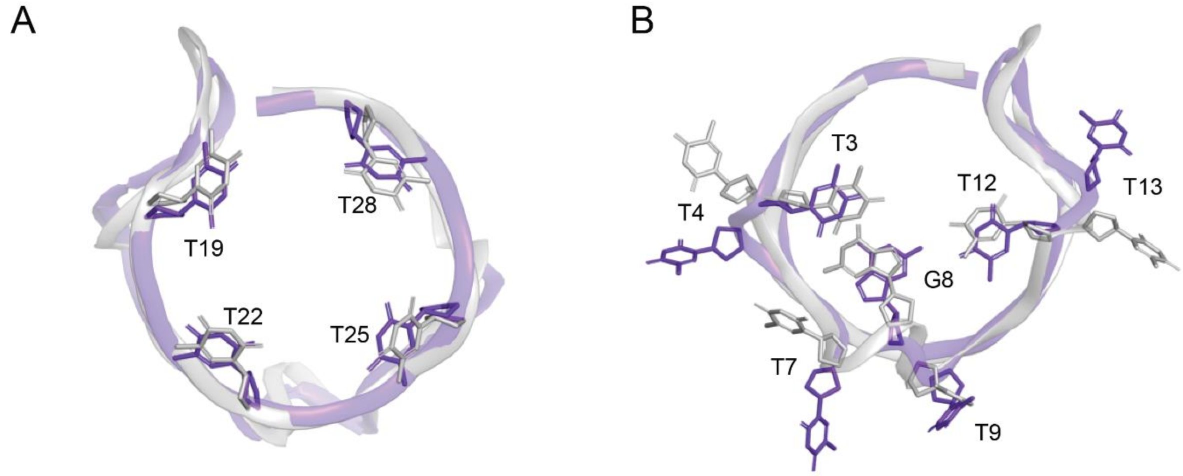

Figure S9. Superposition of the NMR solution (grey) and X-ray crystal (purple) structures. (A) View of the capping bases from the 5’-end of the left-handed block and (B) 3’-end of the right-handed block. The sequence numbering follows that of the *TBA-TT-Block2* sequence used in the NMR structure. Sugars and bases of the G-tetrad core are removed for clarity.

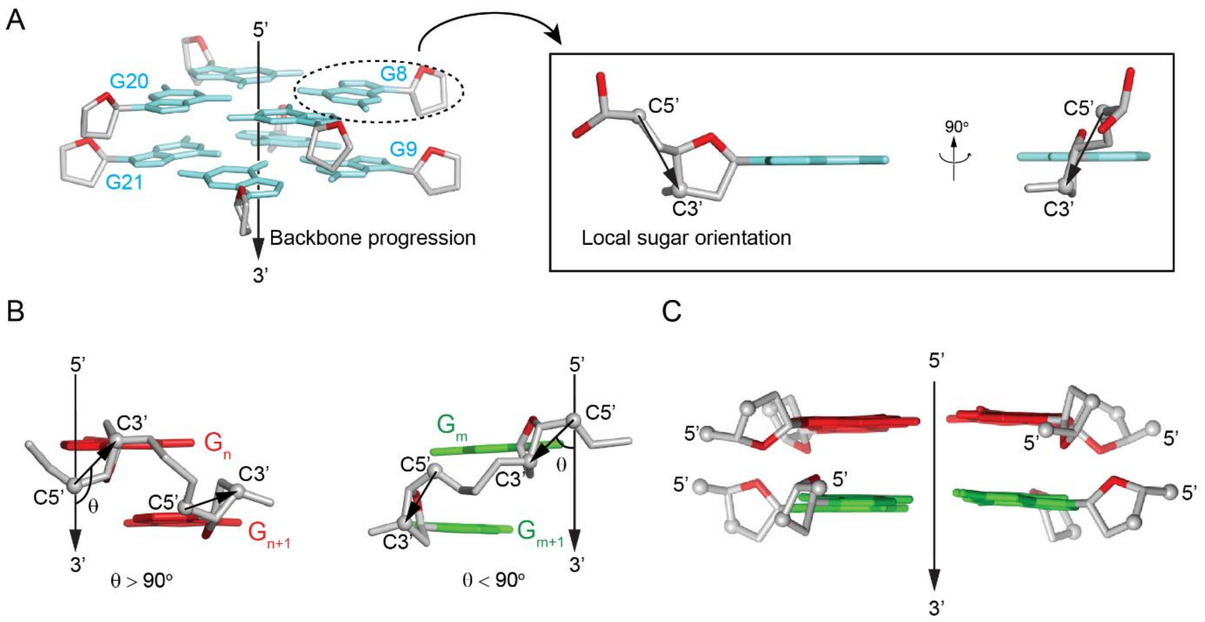

Figure S10. (A) Parallel stacking of two G-tetrads (taken from PDB ID: 1KF1) showing the overall backbone progression orientation from 5' to 3'. In the box is a zoomed-in visualization of a single nucleotide showing the detailed local sugar orientation (from C5' to C3') in two different view angles. (B) Detailed view of angles between local sugar and overall backbone orientations (Ɵ) of a GG-step from *Block2* (left) and *TBA* (right). The angles are shown to be more than 90° and less than 90° for the GG-step of *Block2* and *TBA*, respectively. (C) The stacking interface of *TBA* and *Block2*. The two blocks are shown to have the same overall backbone progression and yet having a 5'-5' sugar stacking mode.

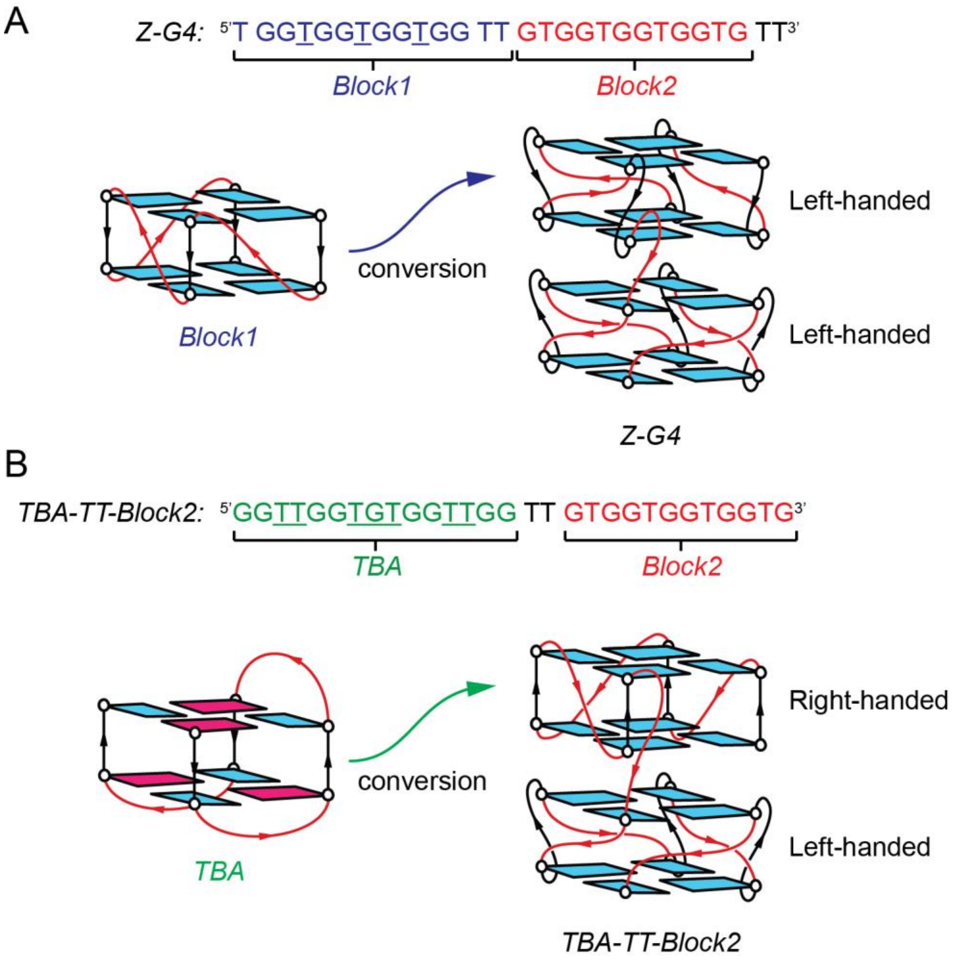

Figure S11. Conformational conversion of (A) *Block1* and (B) *TBA* following attachment of *Block2* on the 3'-end. The sequences, original folding topologies, and final folding topologies are shown. The G4 loops in both *Block1* and *TBA* are underlined. *Block1*-*Block2* (*Z-G4*) adopts a full left-handed folding topology while *TBA-TT-Block2* adopts a right- and left-handed hybrid folding topology.

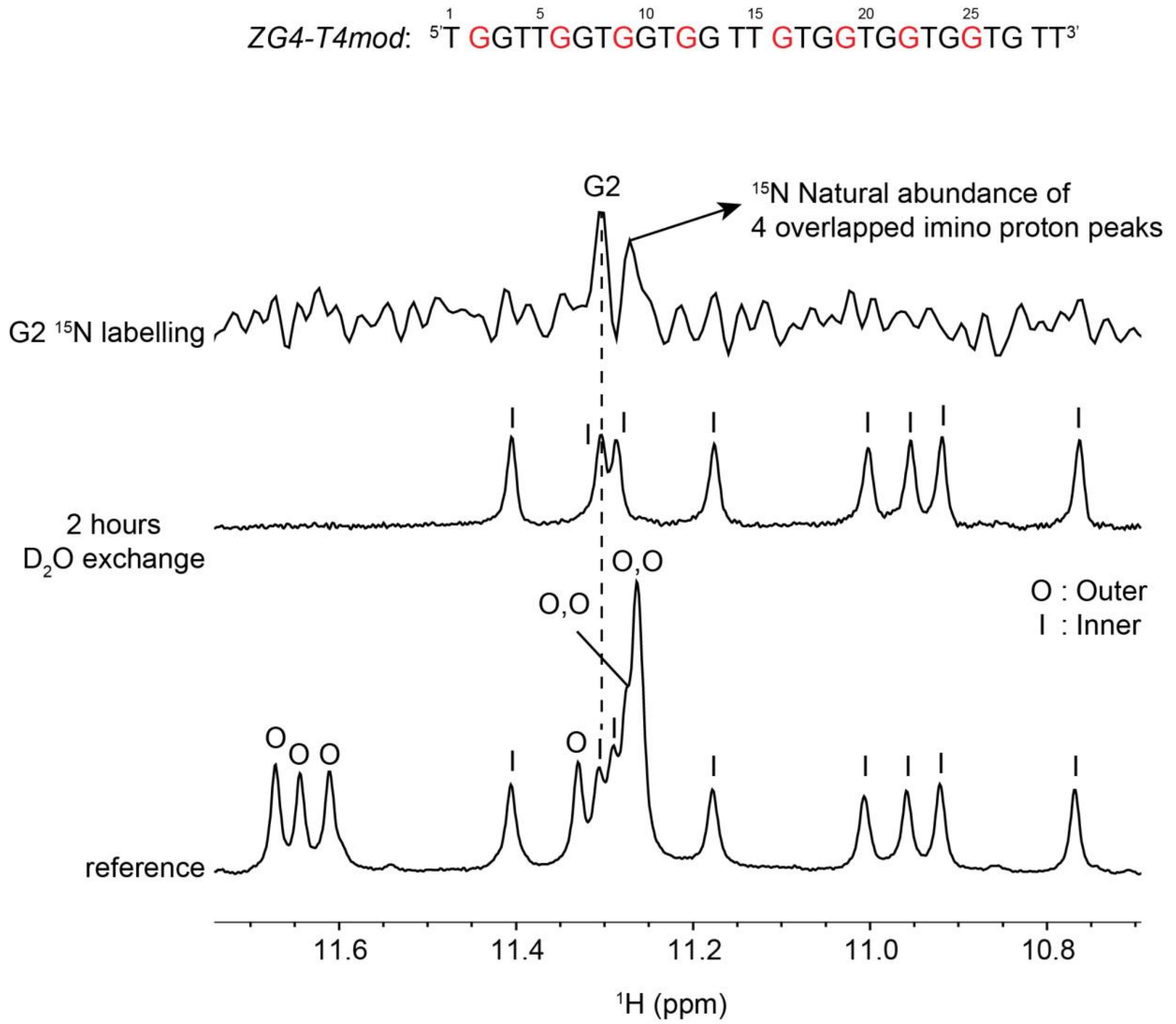

Figure S12. Examination of the *Z-G4-T4mod* sequence with NMR spectroscopy. The sequence is displayed with the inner tetrad guanines indicated in red. The reference, D_2O exchange and the ^{15}N-HMQC spectra are shown (from bottom to top). The D_2O exchange spectrum allowed the determination of the imino proton peaks of inner tetrad guanines. The ^{15}N-HMQC spectrum revealed the imino proton peak of the first 5' guanine (G2). Together, the results showed that G2 is located in the inner tetrad in *Z-G4-T4mod* structure, analogous to the right- and left-handed hybrid structure of *TBA-TT-Block2*.

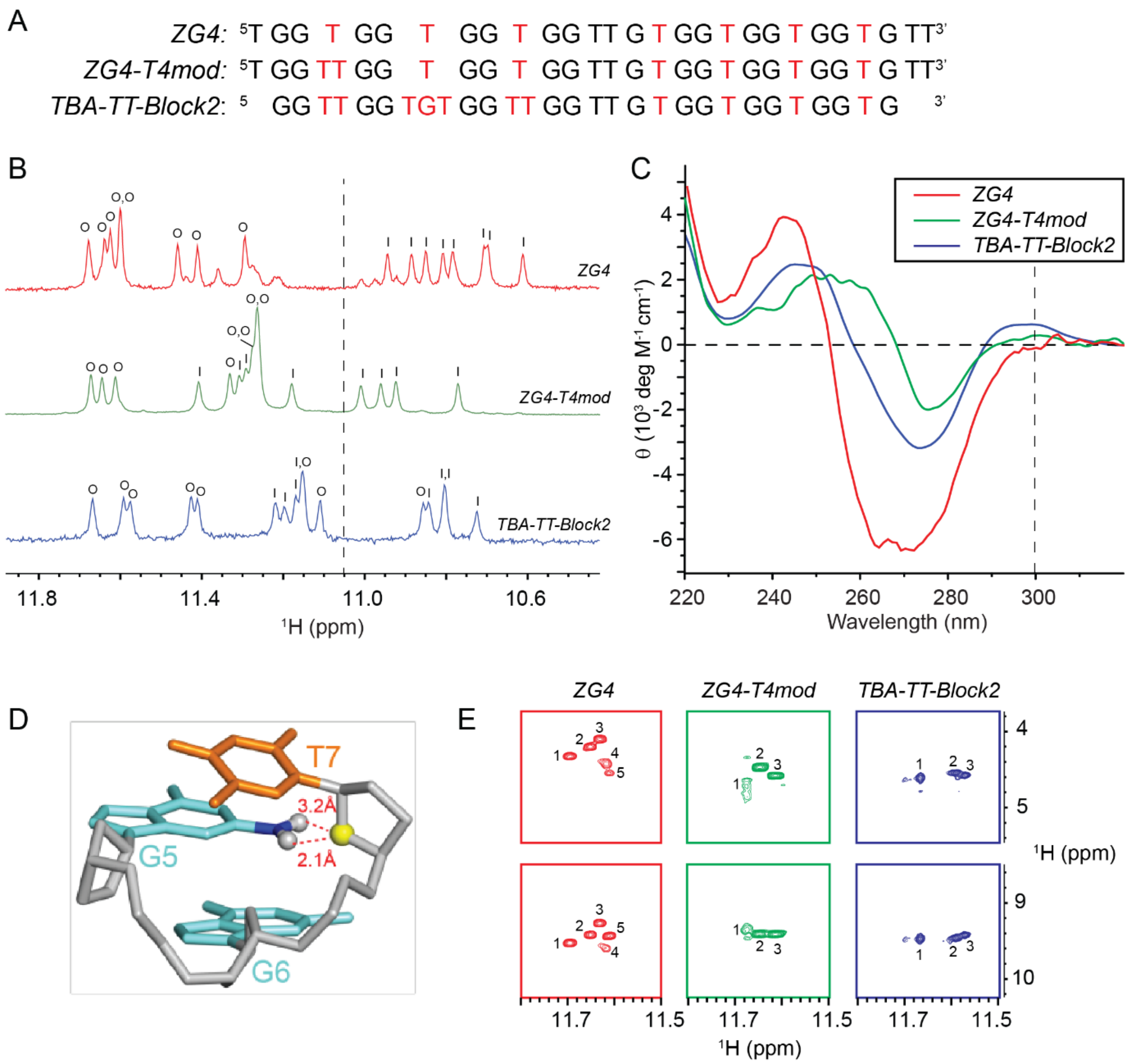

Figure S13. Comparison between *Z-G4, Z-G4-T4mod* and *TBA-TT-Block2* sequences. (A) Sequences *of Z-G4, Z-G4-T4mod* and *TBA-TT-Block2*, indicated in red are the thymine loops between the G-tracts of the three sequences. (B) NMR spectra of the three sequences, outer imino protons are marked with O, and inner imino protons are marked with I (outer and inner imino protons were identified from D_2O exchange experiments). *Z-G4* showed a clear separation between the two groups, while *Z-G4-T4mod* and *TBA-TT-Block2* do not. (C) CD spectra of the three sequences indicating the existence of a left-handed G4 folding. (Note: NMR and CD spectra of *Z-G4* were measured with a longer incubation time after annealing than in (1) to reduce minor conformations, hence the slightly different spectra). (D) Additional hydrogen bond of guanine amino proton towards O4’ atom of capping thymine, unique to the left-handed G4 block. (E) Sharp guanine imino-to-amino protons NOE cross-peaks observed from NOESY (200 to 300 ms mixing time). *Z-G4* showed five pairs of cross-peaks coming from the two left-handed blocks, while *Z-G4-T4mod* and *TBA-TT-Block2* showed three pairs of cross-peaks coming from only one left-handed block.

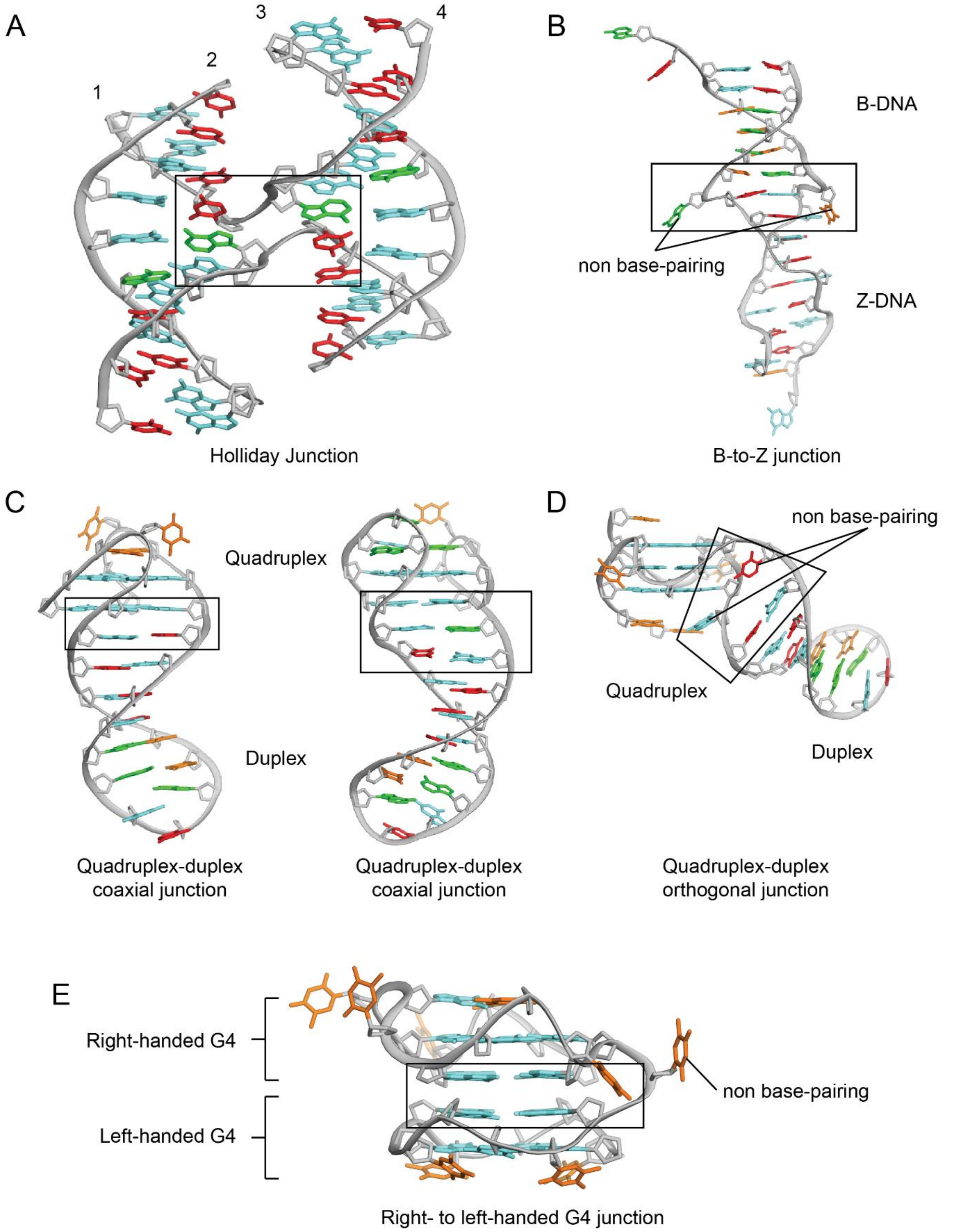

Figure S14. Different structures of helical junctions: (A) Holliday junction (PDB ID: 467D), (B) B-to-Z junction (2ACJ), (C) coaxial (2M8Z and 2M91) and (D) orthogonal (2M93) quadruplex-duplex junctions, and (E) right- to left-handed G-quadruplex (6QJO) junction. Indicated in boxes are the junctions, non base-pairing residues among the junctions are indicated. The nucleobase types are color coded: green indicates adenine; orange thymine; cyan guanine; red cytosine.

Table S1. Backbone dihedral angles

Backbone dihedral	*Block2*	*TBA*	Regular left-handed block (Z-G4)	Regular right-handed block (1KF1)
ε	(327 ± 3)°	(180 ± 21)°	(316 ± 2)°	(197 ± 9)°
ζ	(78 ± 2)°	(271 ± 13)°	(86 ± 3)°	(250 ± 10)°
α+1	(183 ± 5)°	(189 ± 18)°	(161 ± 6)°	(309 ± 17)°
β+1	(221 ± 4)°	(218 ± 27)°	(226 ± 5)°	(180 ± 8)°
γ+1	(41 ± 4)°	(30 ± 21)°	(54 ± 3)°	(33 ± 11)°

Average values in the left-handed block (*Block2*) is obtained from two dinucleotide steps G22-G23 and G25-G26; corresponding values for the right-handed block (*TBA*) is obtained from four dinucleotide steps G1-G2, G5-G6, G10-G11 and G14-G15.

References

1. Chung, W.J., Heddi, B., Schmitt, E., Lim, K.W., Mechulam, Y. and Phan, A.T. (2015) Structure of a left-handed DNA G-quadruplex. *Proc Natl Acad Sci U S A*, **112**, 2729-2733.
2. Phan, A.T. and Patel, D.J. (2002) A site-specific low-enrichment ^{15}N,^{13}C isotope-labeling approach to unambiguous NMR spectral assignments in nucleic acids. *J Am Chem Soc*, **124**, 1160-1161.
3. Phan, A.T. (2000) Long-range imino proton-13C J-couplings and the through-bond correlation of imino and non-exchangeable protons in unlabeled DNA. *J Biomol NMR*, **16**, 175-178.
4. Bakalar, B., Heddi, B., Schmitt, E., Mechulam, Y. and Phan, A.T. (2018) A Minimal Sequence for Left-Handed G-Quadruplex Formation. *Angew Chem Int Ed Engl*, **58**, 2331-2335.
